# Rapid human movement and dengue transmission in Bangladesh: a spatial and temporal analysis based on different policy measures of COVID-19 pandemic and Eid festival

**DOI:** 10.1186/s40249-024-01267-4

**Published:** 2024-12-26

**Authors:** Jahirul Islam, Wenbiao Hu

**Affiliations:** Ecosystem Change and Population Health Research Group, Centre for Immunology and Infection Control, School of Public Health and Social Work, Queensland University of Technology, Kelvin Grove, Brisbane, QLD 4059 Australia

**Keywords:** Dengue transmission, Human movement, Disease surveillance, Infectious disease early warning system

## Abstract

**Background:**

Rapid human movement plays a crucial role in the spatial dissemination of the dengue virus. Nevertheless, robust quantification of this relationship using both spatial and temporal models remains necessary. This study aims to explore the spatial and temporal patterns of dengue transmission under various human movement contexts.

**Methods:**

We obtained district-wise aggregated dengue incidence data from the Management Information System, Directorate General of Health Services of Bangladesh. The stringency index (SI), along with eight individual policy measures (from the Oxford Coronavirus Government Response Tracker database) and six mobility indices (as measured by Google's Community Mobility Reports) were obtained as human movement indicators. A multi-step correlative modelling approach, including various spatial and temporal models, was utilized to explore the associations of dengue incidence with the SI, fourteen human movement indices and the Eid festival.

**Results:**

The global Moran’s *I* indicated significant spatial autocorrelation in dengue incidence during the pre-pandemic (Moran’s *I*: 0.14, *P* < 0.05) and post-pandemic periods (Moran’s I: 0.42, *P* < 0.01), while the pandemic period (2020–2022) showed weaker, non-significant spatial clustering (Moran’s *I*: 0.07, *P* > 0.05). Following the pandemic, we identified the emergence of new dengue hotspots. We found a strong negative relationship between monthly dengue incidence and the SI (*r*_spearman_: − 0.62, *P* < 0.01). Through the selection of an optimal Seasonal autoregressive integrated moving average model, we observed that the closure of public transport (β = − 1.66, *P* < 0.10) and restrictions on internal movement (β = − 2.13, *P* < 0.10) were associated with the reduction of dengue incidence. Additionally, observed cases were substantially lower than predicted cases during the period from 2020 to 2022. By utilising additional time-series models, we were able to identify in 2023 a rise in dengue incidence associated with the Eid festival intervention, even after adjusting for important climate variables.

**Conclusions:**

Overall, rapid human movement was found to be associated with increased dengue transmission in Bangladesh. Consequently, the implemention of effective mosquito control interventions prior to large festival periods is necessary for preventing the spread of the disease nationwide. We emphasize the necessity for developing advanced surveillance and monitoring networks to track real-time human movement patterns and dengue incidence.

**Graphical Abstract:**

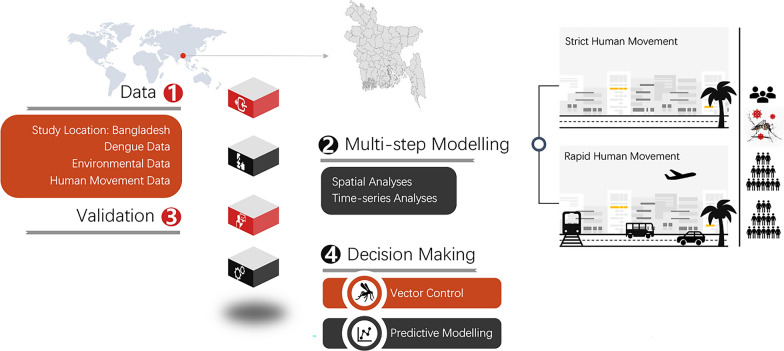

**Supplementary Information:**

The online version contains supplementary material available at 10.1186/s40249-024-01267-4.

## Background

An unprecedented peak of dengue transmission has been observed around the world during 2023 as globally, over 5 million cases are reported from 80 World Health Organization (WHO) regions with 5000 fatalities, leading to an emergency appeal for the year 2024 [1]. Following the remarkable dengue epidemic in 2019, most countries observed a dramatic shift during the COVID-19 pandemic while the majority reported declined incidence rates [2]. On occasion, this is attributed to the underreporting or the influence of restricted human movement [2]. Human mobility and transmission of infectious diseases are interconnected [3]. Mobility is a key aspect of human activity, reflecting behavioural ecology either within a country or across borders encompassing migration, travelling, and displacement on a larger or smaller scale [4, 5]. Significant temporary or swift movement may arise from festival events, societal issues (trade and tourism, job opportunities, rapid urbanization, political unrest), public assemblies, and an array of other influences including those driven by the impact of climate change [6]. The risk of transmitting infectious diseases poses a threat due to the dramatic shift in global and regional interconnectivity, leading to the geographical circulation of viruses into previously unaffected regions [7]. Dengue virus has historically managed to travel long distances from India to the Pacific Islands during the first half of the twentieth century, following the isolation of dengue viruses (DENV1 and DENV2) in Japan in 1943 and Hawaii in 1945 [8]. Dengue transmission is unable to spread through saliva, sexual contact or respiratory droplets; rather, infected individuals may function as hosts for the virus, subsequently infecting female mosquitoes seeking blood meals [9]. Despite 75% infected persons being asymptomatic, a viremic human travelling to other areas poses a risk to the local establishment of this virus, as symptoms may appear after an incubation period of 4–7 days (up to 14 days) [10, 11].

The habitat of dengue mosquitoes is predominantly found in urban areas where both climatic and environmental conditions favour breeding. This occurs particularly in situations involving standing water, which can result from prolonged drainage system blockages, artificial containers, and rooftop gardens [12, 13]. This intricate vector development process may potentially have a broader impact due to the increased proximity between individual and their habitats. Despite the presence of different *Aedes* vector control measures including vaccine development, larval management, the establishment of a viremic surveillance system, and the introduction of *Wolbachia*, the overall number of dengue cases has increased tenfold in 2019 compared to 2000 [14–17]. The rising number of dengue infections among travellers who have visited dengue-endemic countries is raising the alarm for future geographical expansion of this vector [18]. Centres for Disease Control and Prevention released a weekly report highlighting a significant increase in travel-associated dengue fever cases in the USA in 2019, with a 168% leap relative to the average annual incidence from 2010 and 2018 [19]. Similarly, such a growing trend is observed in European countries from 2015 to 2019 and Puerto Rico in 2024 [20, 21]. In addition to leisure and commercial travel, various other local factors can contribute to the dengue virus spillovers. For instance, holidays for religious or cultural festivals such as Eid, Puja, Christmas, and New Year’s eve; concerts and political gatherings, can lead to large-scale human mobility and thereby, may facilitate the viral transmission. Eid is celebrated by almost 2 billion Muslim people globally [22]. Two Eid holidays are celebrated annually, known as Eid-ul-Fitr and Eid-ul-Adha, which typically comply to the lunar calendar. Report indicated that approximately 10 million individuals from Dhaka, the capital district of Bangladesh, and 40 million individuals from other districts travel to their hometowns during each Eid festival [23]. The reason is related to the availability of highest public holidays ranged from 3 to 7 days, longest compared to other occasion, due to their cultural significance. Dengue virus may benefit from such large-scale migration, which occurs on an annual basis, as many other Muslim-majority countries are at risk, including Brunei Darussalam, Indonesia, Malaysia, Maldives, and Yemen in Asia; Burkina Faso, Djibouti, Egypt, Somalia, Sudan, and Tanzania in Africa [24].

COVID-19 pandemic significantly influenced human movement, both within countries and across international borders [25]. In 2020, many dengue-endemic countries, such as Bangladesh, reported a notable reduction in the dengue incidence [26, 27]. Conversely, the incidence of cases climbed during the same timeframe in Peru and Singapore, whereas in Thailand, a stable pattern was observed, followed by a 163.19% decrease in cases by 2021 [28–30]. However, comprehending the relationship between dengue transmission and large-scale rapid movement, as well as the implications of stringent movement patterns, necessitates in-depth analysis. We searched the literature and identified few studies utilizing various human movement indicators associated with dengue transmission, including migration and transportation data, call detail records, COVID-19 related public health and social measures (PHSM), and Google’s Community Mobility Reports (GCMR) [26, 31, 32]. Additionally, studies employed house to house tracking of socially structured human movement [33, 34], and recorded individual mobility patterns [35]. We advanced current understanding by incorporating long-term temporal data to analyze the association between dengue transmission and distinct patterns of human movement.

The aim of our study is to analyse the extensive dengue data from Bangladesh, a dengue-endemic nation characterized by high population density. We seek to assess the impact and extent of COVID-19-related restrictions and large-scale intra-district migration during the Eid festival on the dynamics of dengue virus transmission. In our study, rapid human movement refers to large-scale temporary intra-district migration or movement during the Eid festival, which can significantly influence dengue virus transmission dynamics. In contrast, mobility restrictions during the COVID-19 pandemic refer to limited or strict human movement.

## Methods

### Study location, and dengue data source

Bangladesh is situated in South Asia, between approximately 20°34′N and 26°38′N latitude, and 88°01′E and 92°41′E longitude, on the northern coast of the Bay of Bengal. The country covers an area of 148,460 square kilometres (57,320 square miles) and is divided into eight divisions and 64 districts. The total population is 165,158,616, with a population density of 1119 per square kilometre [36].

Bangladesh has a wet and humid tropical monsoon climate, which is characterised by an annual mean temperature of 25.71 ℃ and 2174.10 mm of precipitation [37]. The climate in Bangladesh is influenced by the Indian Ocean Dipole and the El Niño Southern Oscillation on an interannual basis [37]. Dengue was first documented in Bangladesh in the 1960s. The initial outbreak of dengue hemorrhagic fever occurred in the mid-2000s. From 2000 to 2023, 564,772 clinically confirmed dengue cases have been reported [27, 38, 39]. As a dengue-endemic nation, recognised by the WHO, all suspected, probable, and confirmed cases of dengue are systematically reported within the disease notification system [38]. The highest spatial resolution of the available dengue incidence data is at the district level [39]. Due to its substantial annual incidence of dengue cases, we selected this region as the focus of our hypothesis analysis. District-wise, clinically reported daily dengue incidence data collected from August 2019 to December 2023, was combined with monthly incidence data ranging from January 2012 to July 2024 from the Management Information System, Directorate General of Health Services, Bangladesh [39].

### Climate and population data

The analysis of rapid human movement and its impact on dengue incidence focused specifically on the post-pandemic period, particularly during the three consecutive Eid festivals in 2023 and 2024. Climatic variables—daily average temperature (℃), precipitation (mm), and relative humidity—were integrated into the ARDL model for these periods. These data were obtained from the Visual Crossing weather portal (https://www.visualcrossing.com), which allowed us to capture the unique patterns of human movement and their potential association with dengue dynamics during periods of rapid mobility associated with the Eid festivals. We collected the updated population data from the nationally published Preliminary Report on Population and Housing Census (2022), issued by the Bangladesh Bureau of Statistics. Population data from a single point in time were used to calculate the incidence rate for different years [36].

### Human movement indicators

We obtained the PHSM data from the Oxford COVID-19 Government Response Tracker (OxCGRT) project [40]. Eight datasets comprising several confinement measures, including school closures, workplace closures, cancellation of public events, restrictions on public gatherings, closures of public transport, stay-at-home requirements, restrictions on internal movements, and international travel controls, were selected. Additionally, an overall measure of stringency, known as the stringency index (SI), is provided, with values ranging from 0 to 100, where a score of 100 indicates the highest level of stringency. The PHSM indicators were initially recorded in a categorical format and were then assigned a continuous value based on the equation specified by OxCGRT project [40]. The sub-index score values ranged from 0 to 100 and can be interpreted similarly to the SI. These scores were available from January 2020 to December 2022.

We further collected GCMR data for Bangladesh, which provide metrics on the amount of time individuals spend in six distinct types of locations: residential, grocery and pharmacy, workplaces, transit stations, parks, and retail and recreation [26]. The baseline represents the median value for the same weekday over the first 5 weeks of 2020, specifically from January 3rd–February 6th. The GCMR data spans from February 15th 2020–October 15th 2022, expressed as a percentage change from the baseline. Supplementary 1 (Fig. S1–2, p2–9) provides a comprehensive explanation of all the PHSM and GCMR variables, along with figures demonstrating their temporal progress.

COVID-19-related evaluation indicators can serve as proxies for human movement indicators because they often reflect patterns in population mobility and social interaction. Using COVID-19 indicators to understand human movement allows researchers to analyse mobility patterns without relying on direct tracking data. This approach is particularly useful for assessing movement at larger scales, such as between regions, and understanding its impact on disease transmission.

### Statistical data analysis

#### Detecting spatial autocorrelation, cluster, and spatial correlation

Global Moran’s *I* was applied to explore spatial autocorrelation, using Euclidean metrics as the spatial weight to measure the association within the generated distance [41]. Spatial clusters were detected using the SatSCan software, which utilizes Kulldorff's spatial scan statistics by employing spatial cylinders to identify patterns and applied a discrete Poisson model [42]. The model posits that the frequency of events in a particular geographic region conforms to a Poisson distribution, determined by a known population at risk. Given that we used district-wise aggregate cases numbers and population data from the census, a discrete Poisson model using purely spatial scan statistics was performed, incorporating the geographical coordinates (latitude and longitude) of each district to specify unique locations. We utilized a maximum window size that encompassed 50% of the population at risk and employed a circular window shape with 999 replications. To prevent cluster overlap, we adjusted for 10% of the population at risk, particularly for 2023, due to a significantly higher incidence rate compared to other years [43].

We measured the spatial correlation for three different lag periods, using the Spearman correlation to evaluate the relationship between Dhaka–regarded as the dengue epicentre in Bangladesh–and other districts [44, 45].

#### Geographical regression model and Getis-Ord Gi*

We used the projected coordinate system for the Bangladesh zone. The incidence rate during three time periods was log-transformed to ensure a normal distribution. We built a geographically weighted regression (GWR) model, where the dependent variable was normally distributed dengue incidence rate (per 100,000) for the 2019 (pre-pandemic), 2020–2022 (pandemic), and 2023 (post-pandemic) period. The covariate explored was district-wise population density.

GWR characterizes the association between dependent and independent variables using localised models. Each data point is modelled independently and includes only those observations within a defined neighbourhood (kernel), determined by either a fixed distance or an adaptive distance determined by the density of point samples [46]. Points closer to the regression points are assigned greater weight compared to those that are more distant. A GWR model produces β coefficients and various coefficients of variation for each observation in the sample, formally outlined as follows:

$${Ln(y}_{i})={a}_{i0}\sum_{k=1, m}{a}_{ik}{x}_{ik}+ {\varepsilon }_{i}$$

Here, ${y}_{i}$ represents normally distributed dengue incidence rate, ${a}_{ik}$ represents the local coefficients for the effect of dengue across each district, ${x}_{ik}$ represents population density, ${\varepsilon }_{i}$ is the difference between the observed and the predicted incidence rates. In our study, a Gaussian model type was selected, and the number of neighbours was determined through an optimization process aimed at minimizing the Akaike Information Criterion while maximizing the adjusted R-squared value [47]. A bi-square local weighting scheme was employed due to its comparatively better performance than the Gaussian kernel [46].

Standardised residuals were mapped for three periods utilizing the Getis-Ord Gi^*^ statistic within ArcGIS Pro [41]. The Gi^*^ statistic creates a *z*-score with corresponding *P*-values that assess whether the observed spatial clustering is statistically significant. The suitable neighbourhood search threshold of 62.60 km was provided by ArcGIS Pro (Version 3.1.2, Esri Inc., Redlands, CA, USA).

#### Seasonality and trend analysis

We used the seasonal trend decomposition with LOESS (STL) to explore seasonal and trend elements [48]. STL decomposition involves a series of smoothing operations and ensures flexibility in determining the variation in both trend and seasonal components, even when the missing values are present.

#### Analysing the association of human movement indicators with dengue case

A different seasonal autoregressive integrated moving average (SARIMA) model was fitted using the monthly aggregated dengue incidence data ranging from March 2020 to October 2022, aligning with all available human movement indicators. After selecting an optimal model based on lower Bayesian information criterion (BIC), root mean square error (RMSE), and higher *R*^2^, we used the monthly PHSM and GCMR data to explore the association with dengue cases (Supplementary 1, Table S1). Stationarity of the data was ensured by performing square root transformations on the dengue case, PHSM, and GCMR data. The GCMR data was normalized by assuming 100 percent human movement before the pandemic. Therefore, the percentage change was adjusted by adding 100 prior to performing the square root transformation. Model performance was further evaluated using the Box-Jenkins approach, as well as Autocorrelation function (ACF), and Partial autocorrelation function (PACF) plots [49, 50]. Moreover, eight PHSM and six GCMR sub-indices were included in the selected models to explore association with dengue incidence.

#### Measuring the difference between the predicted and observed cases during the COVID-19 pandemic period (2020–2022)

In order to assess the difference between the observed cases during the pandemic period and predicted cases, we fitted a SARIMA model using the monthly aggregated dengue cases ranged from January 2012 to December 2019. The SARIMA model integrates the seasonal component in the autoregressive integrated moving average (ARIMA) model, which includes autoregressive terms (AR), denoted as lower-case *p*; moving average (MA) terms denoted as *q*; and differencing denoted as *d*. To indicate the additional seasonal component, capital *P, D, Q* represent the seasonal AR, seasonal differencing, and seasonal MA terms, consequently. The AR component is an important measure for exploring the association of historical data with itself, while the MA component indicates whether the residuals and the weighted average of the random disturbance terms are correlated with previous observations [51]. We used the IBM statistical package for the social sciences (SPSS) Expert Modeler to identify the appropriate model parameters which is capable of integrating data to automatically choose the most suitable model [52]. The parameters of the SARIMA model (*p*, *d*, *q*, *P*, *D*, *Q*, and *s*) were determined through meticulous selection. The Ljung-Box test was employed to assess the white noise characteristics of the residuals. We fine-tuned the parameters until the residual sequence of the well-fitted model showed characteristics of white noise.

The choice of the optimal SARIMA model was guided by various criteria, such as the stationary coefficient of determination, mean absolute percentage error (MAPE), RMSE, and normalized BIC (Supplementary 1, Table S2). The optimal model should demonstrate the highest degree of stationarity and the lowest MAPE and BIC values. To avoid the influence of seasonality and the trend component in the dengue data, a square root transformation was applied to maintain stationarity (t = − 4.7805,* P* < 0.01). We further addressed the outliers in the data by selecting the additive, seasonal additive, and additive patch to control for issues with outliers affecting single observations, seasonal periods, and one or more consecutive outliers [53]. We assessed multiple models, and the residuals of these models were examined using ACF and PACF plots (Supplementary 1, Fig. S3-6).

#### Ongoing ARIMA and ARDL model analysis

We fitted an ongoing ARIMA model on a 7-day rolling basis to detect the variation between predicted and observed dengue incidence after the Eid festival. We considered three Eid periods in 2023 and 2024, as these periods fell outside the pandemic timeframe. Daily dengue incidence data for the 1st and 2nd Eid in 2023, and the 1st Eid in 2024 were collected for the periods  22nd February 2023 to May 20th 2023, April 30th 2023–July 26th 2023, and February 10th 2024–May 17th 2024, respectively (ranging 60 days before Eid, 28 days after). The auto.arima method in R software was used for this analysis. Initially, 60 days of data were used to predict the next 7 days, and subsequently, data were added on a rolling basis, extending the model to cover four consecutive weeks after each Eid day [54].

Using the same timeframe and data as the ongoing ARIMA model, we conducted an intervention analysis with the Autoregressive distributed lag (ARDL) model, incorporating key climatic factors such as temperature, precipitation, and humidity. We adjusted the lag period of all climatic variables and performed differencing to ensure stationarity, including for the dengue data, before proceeding to the next step. The results of the Augmented Dickey-Fuller test are presented in the Supplementary 1 (Table S3). Selecting the optimal lag is crucial; therefore, we used the VARselect function in R. Vector autoregressive (VAR) models are commonly employed to analyze the interdependencies and dynamic interactions among variables. The VAR() function approximates a VAR(p) model and requires seven arguments. The variable y should be a data matrix or convertible object. The lag order p is specified as an integer. If unknown, VAR() can automatically determine the appropriate lag order. By setting lag.max to the maximum possible integer and ic to the chosen information criterion (Akaike, Hannan-Quinn, Schwarz, or forecast prediction error), the function identifies the optimal lag inclusion. However, we also considered the significance of the lag period in selecting the final model [55]. The optimal lag periods recommended by the VARselect function are illustrated in Supplementary 1 (Table S4). The goodness of fit for the ARDL model was assessed by examining the residuals and plotting the ACF and PACF of the residuals [56].

All analyses were conducted using RStudio (Version 2024.04.2 + 764, RStudio, PBC, Boston, MA, USA), ArcGIS Pro, and SPSS (Version 29, IBM Corp., Armonk, NY, USA).

## Results

### Patterns of dengue incidence and seasonal variation

Between 2012 and 2023, there were 539,683 reported cases of dengue in Bangladesh. The peak incidences occurred in August, averaging 26.09%, and September, averaging 22.44%. In contrast, the lowest incidences were observed in February at 0.06% and March at 0.05%. In 2023, 59.64% of the incidence was reported, while 18.82% occurred in 2019. Dhaka district accounted for 52.6%, 87.19%, 83.10%, 63.18%, and 35.25% of the total incidence from 2019 to 2023, respectively.

**Fig. 1:**
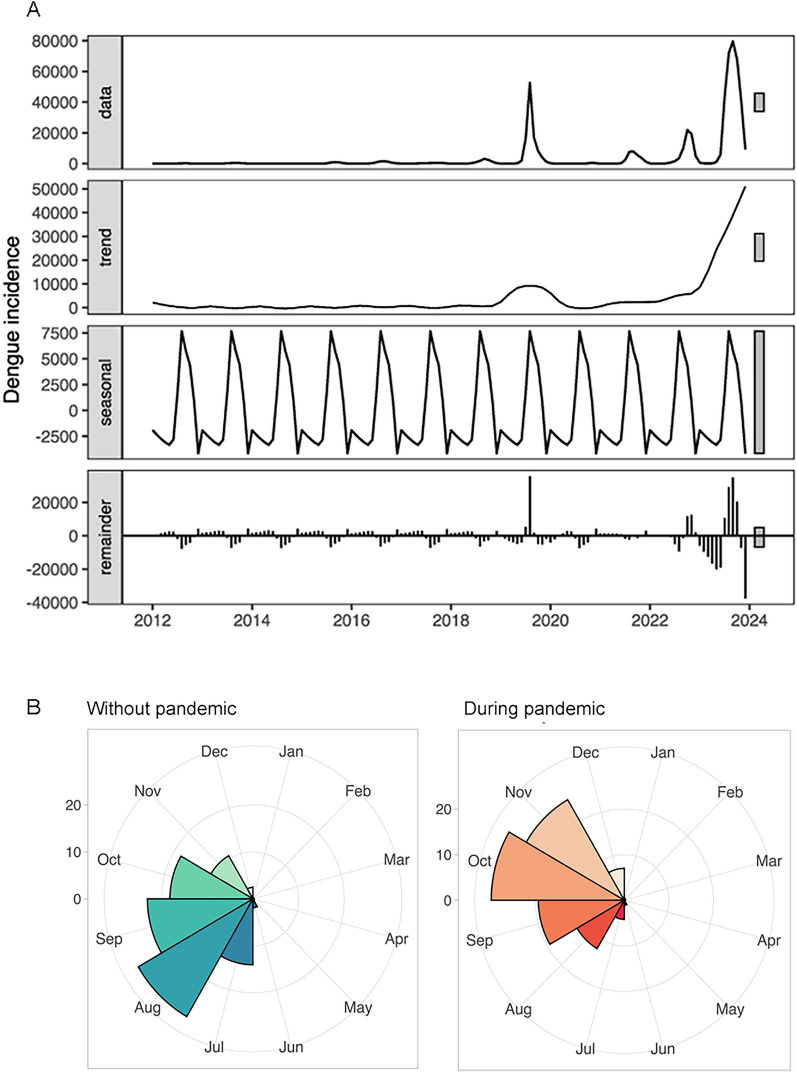
Dengue incidence over time from 2012–2023, including **A** STL decomposition of the monthly dengue incidence from 2012 to 2023, **B** monthly proportion of dengue incidence during the COVID-19 pandemic and the non-pandemic period. The non-pandemic period includes dengue incidence data from 2012 to 2019 and 2023, while the pandemic period covers data from  2020–2022

The STL decomposition indicated seasonality from July to November, peaking in August on a periodic cycle basis. The highest trend was recorded in 2023, with the second peak in 2019, followed by a decline in 2020 and subsequent steady growth afterwards, as illustrated in Fig. 1A. We examined potential changes in seasonality during the pandemic period and identified a significant shift of 2 months through the calculation of monthly case percentages, as illustrated in Fig. 1B.

The incidence of dengue exhibited significant fluctuations, with notable variations observed after 2018. In 2019, there was a yearly increase of 898.76% compared to 2018. This was followed by a decrease of 98.61% in 2020 relative to 2019, and an increase of 417.21% in 2023 compared to 2022.

### Spatial autocorrelation and cluster of dengue incidence

**Fig. 2:**
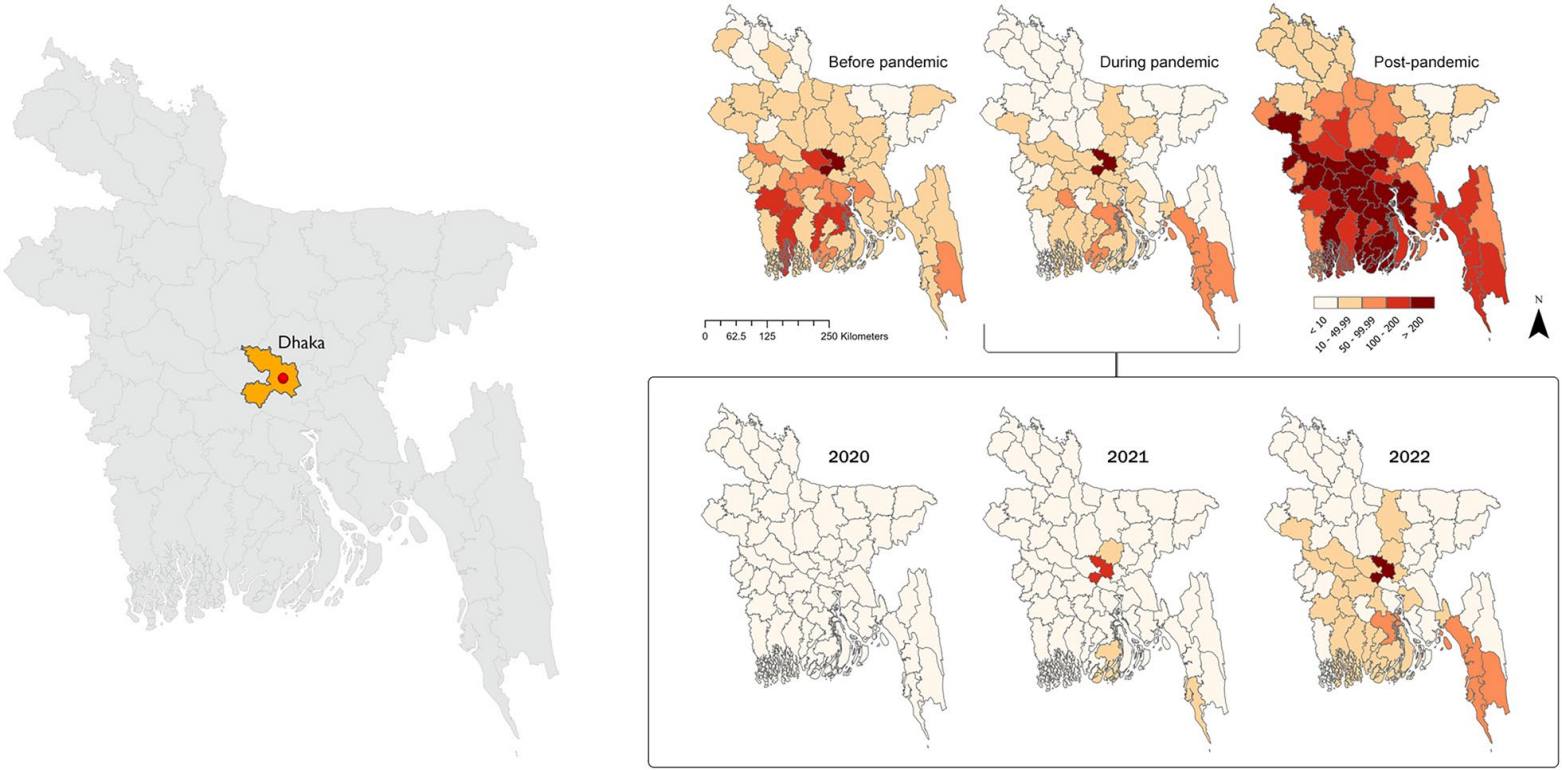
Dengue incidence rate mapping from 2019 to 2023, indicating the risk per 100,000 population. The subnational administrative boundaries of Bangladesh were obtained from the United Nations Office for the Coordination of Humanitarian Affairs [available at: https://data.humdata.org/dataset/cod-ab-bgd?]

The three districts with the highest incidence rates were Dhaka (IR: 361.86), Barishal (IR: 142.15), and Jessore (IR: 132.21) in 2019; Dhaka (IR: 436.13), Cox’s Bazar (IR: 86.21), and Bandarban (IR: 72.54) from 2020 to 2022; and Manikganj (IR: 829.06), Dhaka (IR: 765.76), and Pirojpur (IR: 611.67) in 2023, as showed in Fig. 2.

A lower, but significant, spatial autocorrelation was detected prior to the pandemic, whereas a weakened relationship was identified throughout the epidemic period. In 2023, considerable spatial clustering was observed, as indicated in Table 1.

**Table 1:** Spatial autocorrelation of dengue incidence rate in Bangladesh from 2019 to 2023

Period	Incidence rate (1/100,000)	Moran’s *I*	*z*-score	*P*-value
2019 (Pre-pandemic)	61.37	0.14	2.10	< 0.05
2020–2022 (Pandemic)	55.83	0.07	1.73	> 0.05
2023 (Post-pandemic)	193.55	0.42	4.69	< 0.01

**Fig. 3:**
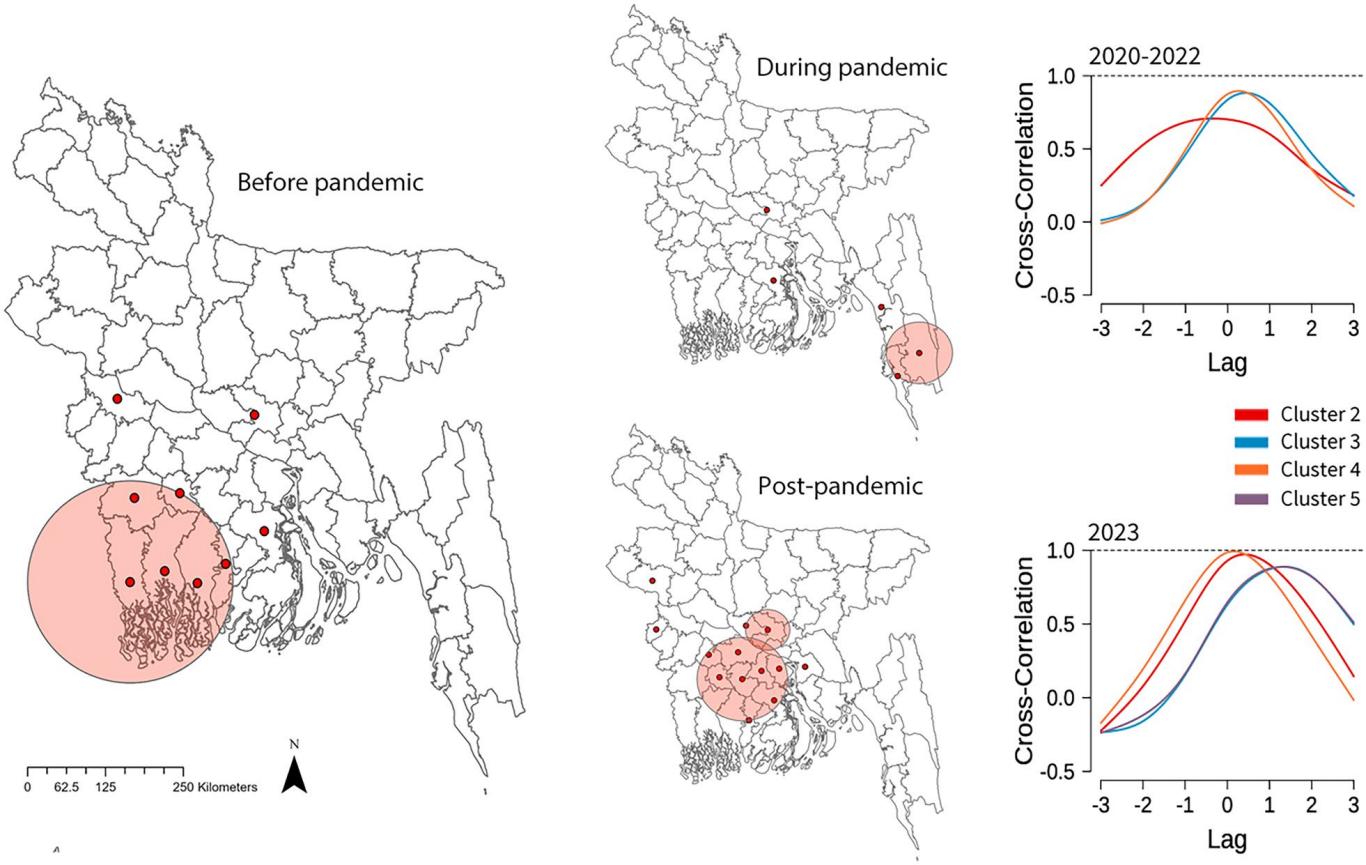
Spatial cluster detection using SaTScan for three time periods from 2019 to 2023 and the monthly lag association between the primary and other clusters. For the cluster analysis, the total yearly incidence was used for the years 2019 and 2023. The pandemic period combines incidence data from 2020 to 2022. The subnational administrative boundaries of Bangladesh were obtained from the United Nations Office for the Coordination of Humanitarian Affairs [available at: https://data.humdata.org/dataset/cod-ab-bgd?]

The discrete Poisson model for purely spatial scan statistics consistently identified Dhaka district as the primary cluster before and during the pandemic period. However, in 2023, both Manikganj and Dhaka districts were identified as primary cluster, as illustrated in Table 2. The spatial scan statistics indicated notable geographic shifts in dengue clusters before, during, and following the pandemic. Dhaka consistently emerged as the principal cluster with the highest risk, particularly during the pandemic when the relative risk (RR) escalated to 23.47. Following the pandemic, Manikganj became part of the major cluster with Dhaka, although the RR decreased to 5.91. Prior to the pandemic, Barishal was recognised as a significant cluster; however, during the pandemic, the second cluster shifted to Bandarban and Cox’s Bazar, exhibiting a declined RR of 1.53. Subsequent to the pandemic, a broad second cluster appeared, comprising several districts such as Gopalganj, Madaripur, Narail, Faridpur, Shariatpur, Barishal, Magura, and Pirojpur, demonstrating a relative risk of 2.31. Furthermore, Meherpur, Chadpur, and Rajshahi emerged as a new cluster in the post-pandemic period, with relative risks of 1.59, 1.08, and 1.07, respectively. These findings underscore the continued prevalence of high-risk regions such as Dhaka and the establishment of new hotspots, such Manikganj and Rajshahi, following the COVID-19 pandemic. All the clusters are illustrated as a map in Fig. 3.

**Table 2:** Clusters of dengue incidence and relative risk (RR) across different time periods during 2019–2023

Clusters	Before pandemic (2019)	RR (95% *CI*)	During pandemic (2020–2022)	RR (95% *CI*)	After pandemic (2023)	RR (95% *CI*)
1	Dhaka	11.33 (11.32, 11.34)	Dhaka	23.47 (23.46, 23.48)	Dhaka, Manikganj	5.91 (5.90, 5.92)
2	Barishal	2.37 (2.34, 2.40)	Bandarban, Cox’s Bazar	1.53 (1.49, 1.57)	Gopalganj, Madaripur, Narail, Faridpur, Shariatpur, Barishal, Magura, Pirojpur	2.31 (2.30, 2.32)
3	Satkhira, Khulna, Bagerhat, Jessore, Pirojpur, Narail	1.44 (1.42, 1.46)	Chittagong	1.16 (1.13, 1.19)	Meherpur	1.59 (1.55, 1.63)
4	Kushtia	1.19 (1.14, 1.24)	Barishal	1.20 (1.15, 1.25)	Chandpur	1.08 (1.05, 1.11)
5	–	–	–	–	Rajshahi	1.07 (1.04, 1.10)

*RR* relative risk; *CI* confidence interval

The incidence rate for each cluster was calculated monthly, and cross-correlation analysis was employed to examine the lag relationship between the primary cluster and all other clusters. The strongest lag relationship was identified at lag 0 during the pandemic, while in the post-pandemic period, the strongest relationship occurred at lag 1 for clusters 3 and 5, as depicted in Fig. 3. A following lag relationship was examined using district-specific incidence rates. The Dhaka district was employed to examine the relationship with other districts. The majority of districts demonstrated a high correlation at lag 0 month both before and after the pandemic. However, the number of districts demonstrating this correlation decreased during the pandemic, as illustrated in Supplementary 1 (Fig. S7).

**Fig. 4:**
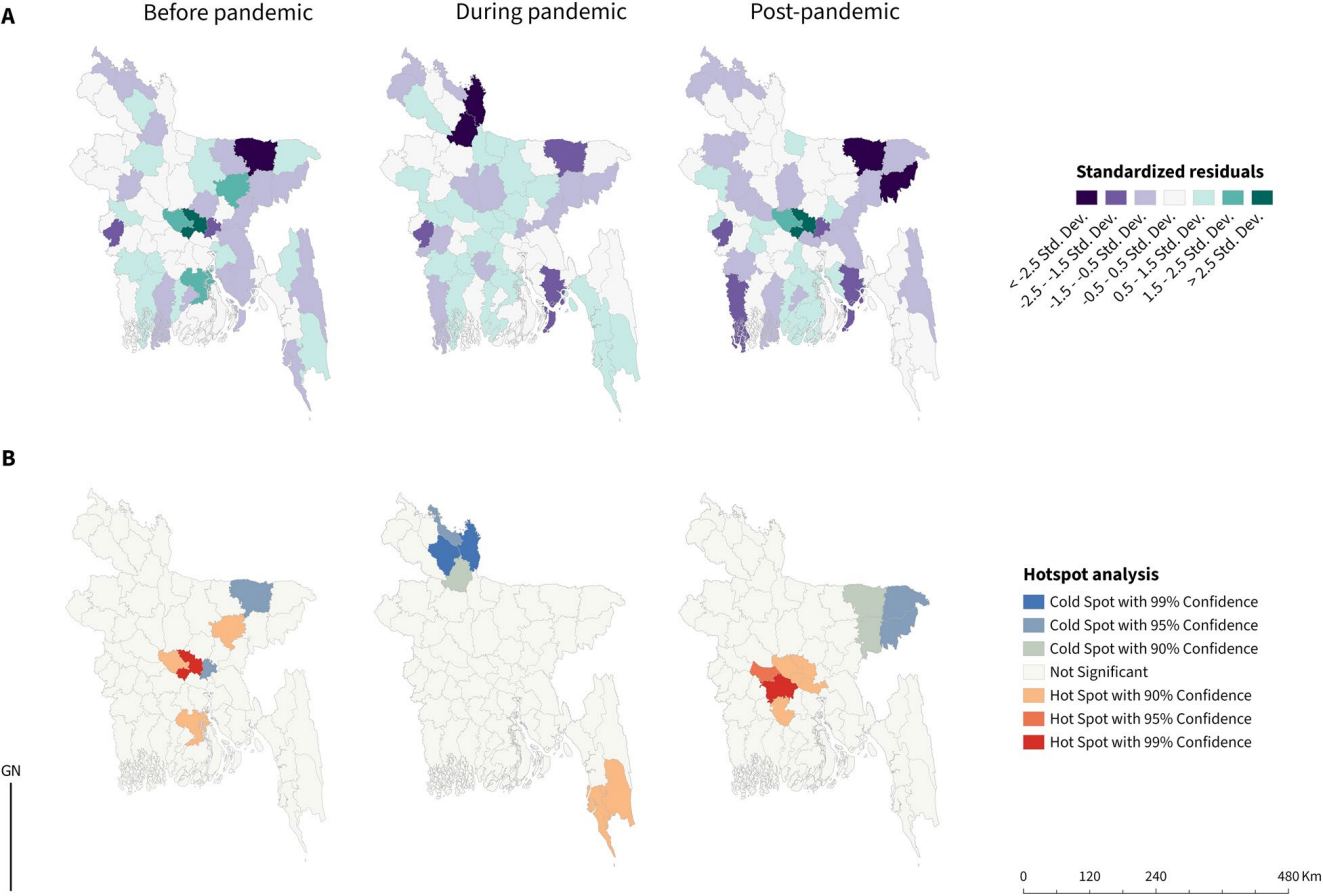
Spatial Analysis of dengue incidence in Bangladesh before, during, and after the COVID-19 pandemic, including **A** Spatial patterns of standardized residuals derived from Geographically Weighted Regression (GWR) for dengue incidence rates, **B** Hotspot analysis of dengue incidence rates using the Getis-Ord Gi* statistic. The subnational administrative boundaries of Bangladesh were obtained from the United Nations Office for the Coordination of Humanitarian Affairs [available at: https://data.humdata.org/dataset/cod-ab-bgd?]

The GWR model produced the maps indicating higher risk in Dhaka, Barishal, Manikganj, Kishoreganj in 2019, Dhaka in the pandemic period, and Dhaka, Manikganj district in 2023, as illustrated in Fig. 4A. Then, using the Getis-Ord Gi* statistic, the standard residuals from the GWR model were utilized to present the spatial distribution of probabilities as being significantly higher or lower than the global mean prediction, with confidence intervals of 90%, 95% and 99%. Predicted hotspots were found in Dhaka, Manikganj, Kishorganj, and Barishal in 2019; Coxs Bazar and Bandarban in the pandemic period; and Faridpur, Rajbari, Dhaka, Manikganj, Gopalganj, Munshiganj in 2023, as illustrated in Fig. 4B.

### Dengue incidence under strict human movement during the pandemic period

**Fig. 5:**
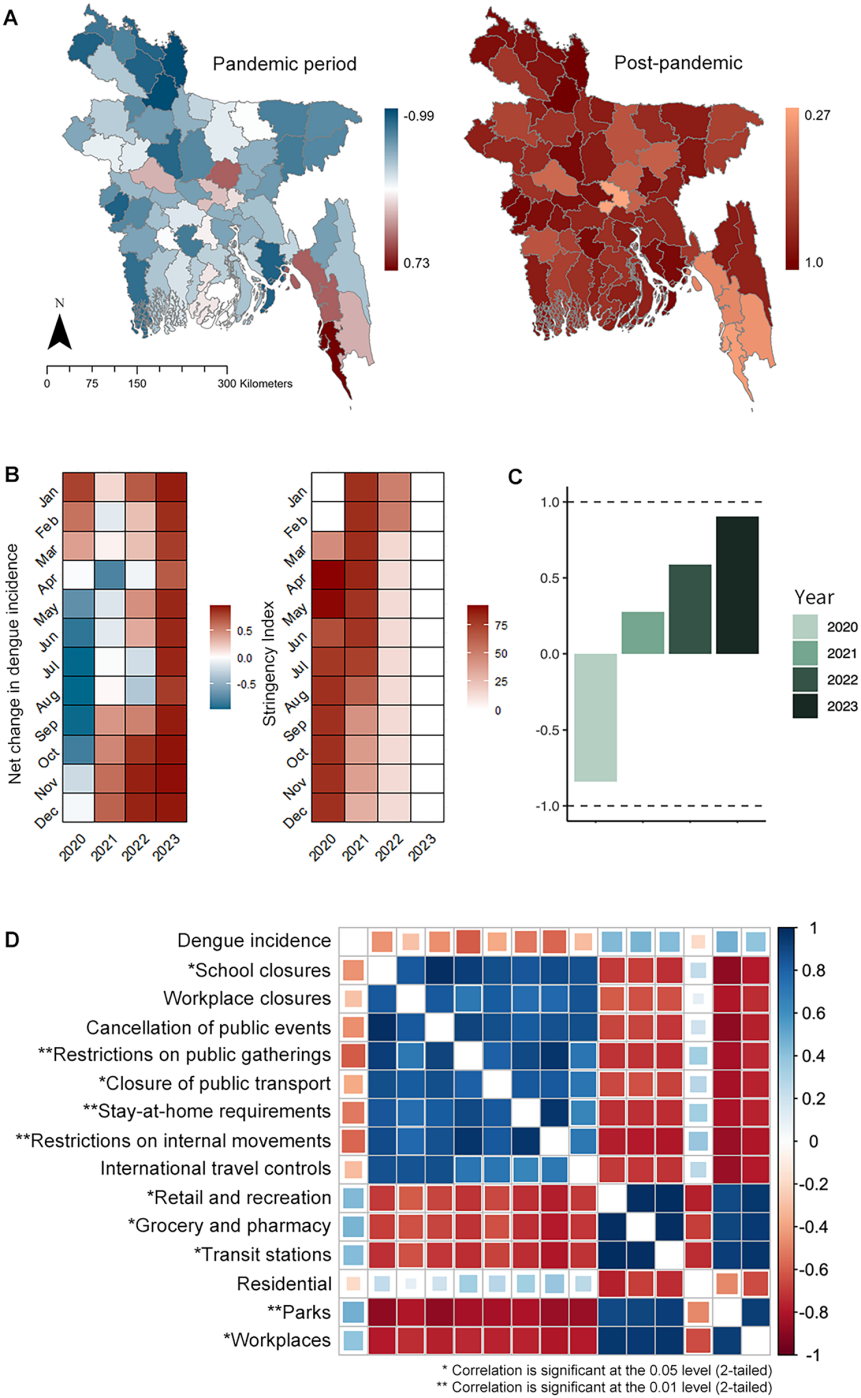
Spatial and temporal net change of dengue incidence and its relationship with PHSM and GCMR, including **A** Net change ratio of annual dengue incidence in 2020–2022 and 2023 versus the incidence of 2019 on a spatial scale, **B** The net change ratio of monthly dengue incidence relative to the baseline period (2012–2019), **C** Yearly net change ratio compared to the baseline period, **D** Correlation plot for the relationship between PHSM and GCMR. Net change: $(C-P)/(C+P)$, where c indicates the spatial/temporal cases in the current period, and P indicates cases before. For example, to calculate the monthly net change for January 2020, the formula will be $(January\, 2020-average\, of\, January\, <span class='convertEndash'>2012-2019</span>/(January\, 2020 +average\, of\, January\, <span class='convertEndash'>2012-2019</span>)$. Net change calculation provides a value ranging from − 1 to + 1. The subnational administrative boundaries of Bangladesh were obtained from the United Nations Office for the Coordination of Humanitarian Affairs [available at: https://data.humdata.org/dataset/cod-ab-bgd?]. *PHSM* public health and social measures; *GCMR* Google’s community mobility reports

We observed a notable reduction in dengue incidence during the pandemic period, with a significantly negative net change ratio across most districts, except for Coxs Bazar (0.73), Chittagong (0.40), Gazipur (0.40), Bandarban (0.14), Pabna (0.13), and Dhaka (0.09). Positive net changes were recorded during 2023, varying between 0.27 and 1 (Fig. 5A). The monthly net change indicated a decline starting from April 2020 and an increase from September 2021 (Fig. 5B). Spearman correlation revealed a negative association between monthly net change and the SI (*r*_spearman_: − 0.62, *P* < 0.01). Notably, the yearly net change showed a pronounced decline in 2020, as visualized in Fig. 5C.

The majority of the PHSM and GCMR indices including school closures (*r* = − 0.37, *P* < 0.05), cancellation of public events (*r* = − 0.43, *P* < 0.05), restrictions on public gatherings (*r* = − 0.58, *P* < 0.01), closure of public transport (*r* = − 0.419, *P* < 0.05), stay-at-home requirements (*r* = − 0.530, *P* < 0.01), restrictions on internal movements (*r* = − 0.57, *P* < 0.01), movement in retail and recreation places (*r* = 0.39, *P* < 0.05), grocery and pharmacy (*r* = 0.39, *P* < 0.05), transit stations (*r* = 0.41, *P* < 0.05), parks (*r* = 0.50, *P* < 0.01), and workplaces (*r* = 0.39, *P* < 0.05) showed a significant relationship with dengue incidence, as illustrated in Fig. 5D.

Following covariate selection, we employed the SARIMA (2,0,1) × (0,1,0)_12_ model, according to model selection metrics. The cancellation of public events (β = 1.02, *P* < 0.10), closure of public transport (*β* = − 1.66, *P* < 0.10), and restrictions on internal movement (*β* = − 2.13, *P* < 0.10) had a significant association with dengue incidence, as mentioned in Table 3. The model residual plots are illustrated in Supplementary 1 (Fig. S8-12).

**Table 3:** SARIMA model results for various human movement sub-indices

${(p,d,q)\times (P,D,Q)}_{12}$	Estimate	SE	*t*	*P*–value
School closures	0.95	0.97	0.98	0.34
Cancellation of public events	1.02	0.56	1.83	0.09
Restrictions on public gatherings	0.54	1.27	0.43	0.68
Closures of public transport	− 1.66	0.94	− 1.78	0.09
Stay-at-home requirements	2.22	2.45	0.91	0.38
Restrictions on internal movements	− 2.13	1.18	− 1.81	0.09
Retail and recreation	− 0.32	3.98	− 0.08	0.94
Grocery and pharmacy	− 0.77	6.31	− 0.12	0.91
Transit stations	− 0.76	3.19	− 0.24	0.81
Parks	− 1.63	3.35	− 0.49	0.63
Workplaces	0.56	2.51	0.22	0.83

*SE* standard error

**Fig. 6:**
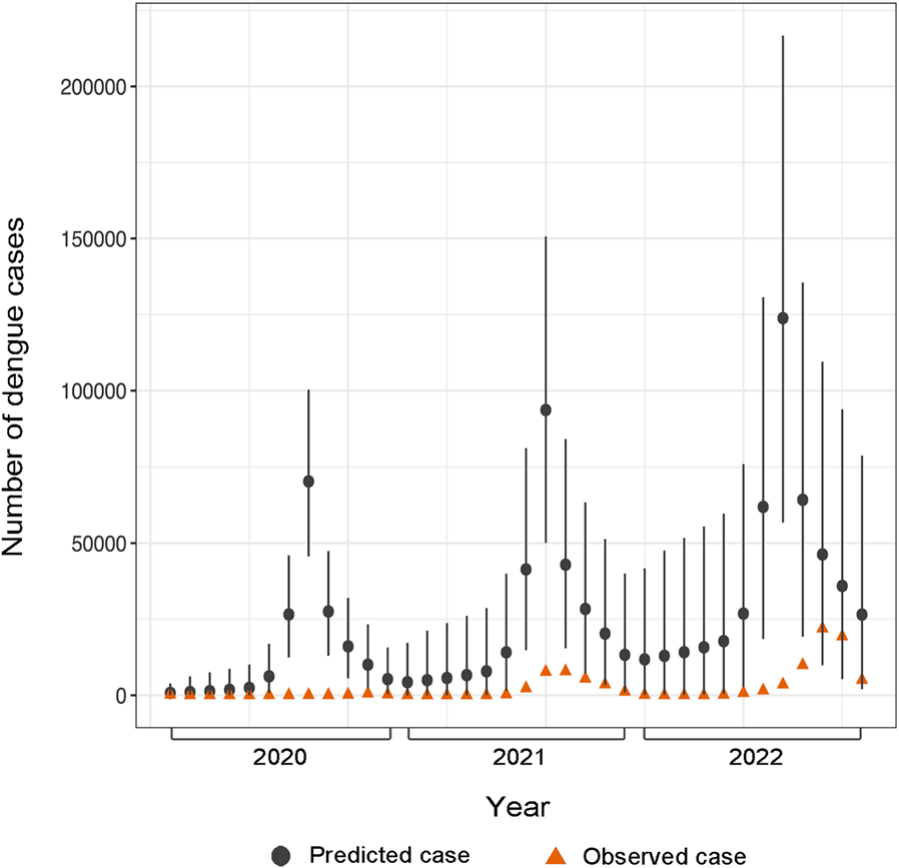
The difference between the predicted and observed cases during the pandemic period (2020–2022) was estimated using the SARIMA (2,1,1) × (0,1,0)_12_ model. Monthly dengue cases from 2012 to 2019 were used to make predictions for 2020–2022. *SARIMA* seasonal autoregressive integrated moving average

To predict the monthly incidence for the pandemic period based on the historical data, we selected SARIMA (2,1,1) × (0,1,0)_12_ based on the model performance metrics, and we found that the predicted cases were substantially higher than the observed cases, as illustrated in Fig. 6. Fitted models and residuals are provided in Supplementary 1 (Table S1).

### Influence of Eid festival on dengue incidence

**Fig. 7:**
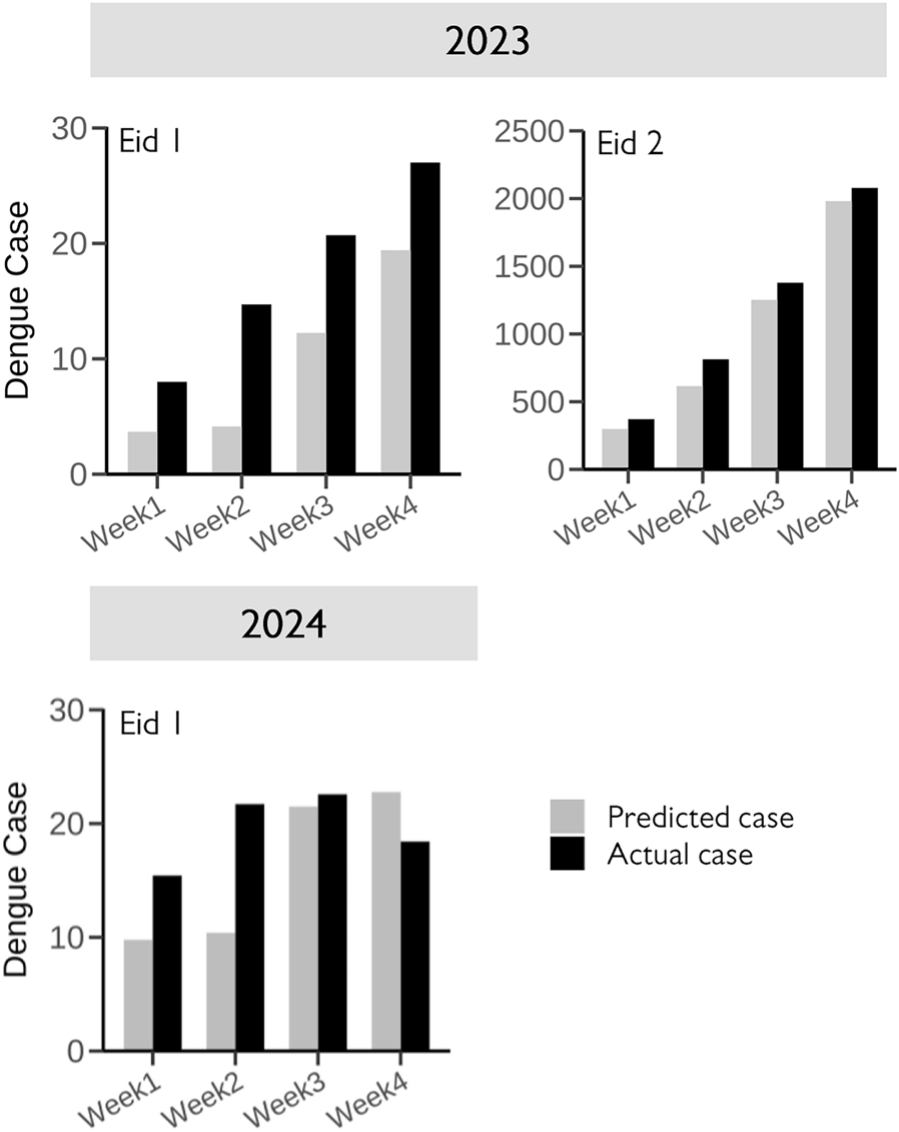
Ongoing ARIMA model and the difference between observed and predicted dengue cases following the Eid festival. An ARIMA model was fitted using the auto.arima method in R software. *ARIMA* autoregressive integrated moving average

The ongoing ARIMA model generated weekly prediction scenarios over a 4-weeks prediction horizon, demonstrating occasions in which the observed case counts exceeded the projected values, as depicted in Fig. 7. The residuals are shown in the Supplementary 1 (Fig. S13-15). The ARDL model demonstrated a positive impact of the Eid festival on dengue incidence, particularly for Eid 1 (*β* = 4.05, *P* < 0.01), and Eid 2 (*β* = 20.23, *P* < 0.01) in 2023. However, Eid 1 in 2024 (*β* = 2.71, *P* > 0.05) did not show a statistically significant effect (Table 4). The model’s goodness of fit, extended results, and lag selection are illustrated in Supplementary 1 (Fig. S16-18, Table S5).

**Table 4:** The parameters of selected ARDL models used during the two Eid periods

Eid festival	ARDL model	Coefficient	*t*-statistic	*P*–value
Eid 1 (2023)	ARDL (6,1,1,4)	4.05	2.77	< 0.01
Eid 2 (2023)	ARDL (10,2,1,8)	20.23	4.05	< 0.01
Eid 1 (2024)	ARDL (6,1,1,4)	2.71	1.27	> 0.05

*ARDL* autoregressive distributed lag

## Discussion

By combining the most updated and longest dengue database available for Bangladesh, we provided empirical evidence to understand the role of rapid and strict human movement on dengue. We showed that the temporal and spatial patterns of dengue transmission varied during the pandemic period, with non-significant spatial autocorrelation (Global Moran’s *I* = 0.07, *P* > 0.05). We found that the closures of public transport and restrictions on internal movements were associated with a decline in dengue incidence. Additionally, we identified averted cases during the pandemic period and a resurgence after the Eid festival.

During the pandemic period, Bangladesh experienced a shift in the seasonality pattern of dengue, with increased incidence observed in October and November. This raises concerns regarding whether dengue is transitioning to a year-round infection pattern or if this change is attributable to recent weather variability. While this monthly cycle coincides with travel-associated dengue cases observed in returning travellers from Southeast Asia, further understanding is necessary [57]. Following the prolonged shutdown in 2020, travel restrictions were gradually lifted starting in September 2021 (Fig. 5B), leading to a corresponding increase in human movement. This change impacted typical travel behaviours and business opportunities and was accompanied by a gradual increase in dengue incidence. Dhaka district remains the primary hotspot in Bangladesh, characterized by the highest relative risk and incidence rate. In dengue-endemic countries, major cities are typically more affected due to higher population density, microclimatic conditions, and rapid mobility [17]. However, rural regions may face increased risk in the future due to development-driven human movement [58]. Consequently, developed districts in Bangladesh, including the identified hotspots, should receive greater attention in terms of strategic and policy interventions to reduce transmission intensity from the epicentre.

Our findings are consistent with those reported by Chen et al., who identified a relationship between COVID-19 pandemic-related human movement indicators and dengue incidence [26]. Furthermore, restrictions on human movement may lead to a decrease in plastic usage, an increase in time available for cleaning rooftop water storage, and a reduction in visits to retail shops or playground gatherings- factors that are recognized as risk factors for *Aedes* mosquitos in an urban setting [59]. The closures of major travel locations may have resulted in a reduction of imported cases, resulting in predominantly local transmission [60]. While the possibility of underreporting dengue incidence cannot be dismissed, the number of deaths should reflect a typical trend, raising scepticism regarding the extent of underreporting of dengue incidence in 2020. Nonetheless, it is clear that human movement is a critical variable that should be incorporated into future predictive models.

The multi-step modelling approach employed in this study provides further evidence of the influence of the Eid festival on dengue incidence. This finding is consistent with another study that demonstrated a relationship between festival-related activities and the escalation of dengue cases [61]. To our knowledge, quantifying the association of human movement and dengue incidence using various robust modelling approaches is essential. Several reasons justify the use of the ARDL model alongside ARIMA. ARDL models offer flexibility in integration orders and can simultaneously analyze short- and long-run relationships simultaneously. They are suitable for smaller sample sizes, address collinearity effectively, facilitate optimal lag selection, and enable cointegration testing while balancing model complexity with explanatory power [62].

We employed a dataset comprising 88 days of dengue incidence, temperature, precipitation, and humidity data in the ARDL model, which resulted in a relatively small sample size. We identified a strong lagged association between dengue incidence and climatic variables. Consequently, prior to conducting the ARDL model-based analysis, we determined the optimal lag period for each variable and performed unit root tests to ensure stationarity. Thus, the ARDL model served as a crucial tool for assessing the impact of the Eid intervention on dengue incidence.

An important potential application of our approach is to aid in predicting and responding to increases in disease incidence that may occur during upcoming festivals or mass movement events. Consequently, several control measures can be implemented in a timely manner to prevent outbreaks in novel or previously unaffected regions. For instance, in Panadura, where 60% of the dengue cases in Sri Lanka are reported, workers were employed to eliminate vector breeding sites after identifying larval locations. This method has proven to be cost-effective in terms of cost per disability-adjusted life year averted and has demonstrated positive results within a few weeks [63]. Similarly, a study conducted in Hangzhou, China, illustrated the rapid effectiveness of vector control measures implemented shortly after their introduction [64]. Such government initiatives may contribute to a reduction in incidence until the availability of mass vaccination or successful evidence-based *Wolbachia* interventions [15, 16, 65].

Concerning the human movement sub-index, the OxCGRT encompasses data from 150 countries; however, only a limited number provide sub-national level policy measures. For Bangladesh, only temporal data was accessible on a daily basis. The GCMR data, another critical indicator of human movement utilized in this study, was also available within a temporal framework. The use of PHSM and GCMR has been extensive throughout the pandemic, demonstrating that the global scope, depth of policy information, and organized structure of this data have significantly contributed to various research domains, including the assessment of their impact on infectious diseases [26, 66–68]. Most importantly, the fourteen sub-indices employed in this study offer nuanced insights into the dynamics of human movement in real-world contexts. Notably, school closures exhibited one of the strongest associations with decreased dengue transmission. Research has demonstrated that school closures are linked to a significant reduction in dengue risk, particularly in the short term (0–1 month), with a lesser impact observed over the longer term (3 months). For instance, in Sri Lanka, periods of school closure in 2021 were found to correlate negatively with dengue cases [26, 69]. While our study identified that indicators such as the closures of public transport and restrictions on internal movements- aside from school closures-are significant, all indices may reflect the increased exposure of individuals to *Aedes* vectors. During the pandemic, the reduction or delay of vector control measures likely contributed to an increased mosquito population. This escalation, in turn, may have intensified human-vector interactions within residential areas, thereby facilitating an increase in dengue incidence despite reduced human mobility [70]. At the community level, socio-economic and demographic factors further influence varying levels of exposure to dengue. Additionally, immunity or serological status, which can differ across regions, may also play a critical role in these dynamics.

The typical flight range of *Ae. aegypti* mosquitoes is approximately 50–100 metres. However, the virus incubation period within humans, which can span several days, facilitates transmission over longer distances [71]. This is consistent with findings from a study conducted in Bangkok, Thailand, where public transport networks were identified as key factors influencing the spatial distribution of dengue [72]. Public transport facilitates widespread human movement across the urban areas, potentially exacerbating the spread of dengue. A study from Cornell University further corroborated this by revealing an association between higher dengue incidence and the proximity of public transportation in economically disadvantaged regions [73]. These findings underscore the intricate link between human mobility and dengue transmission, highlighting the necessity of integrating comprehensive human movement data into future dengue prediction models. Moreover, employing robust spatiotemporal models that incorporate big data such as climate variables, human movement, and festival-related activities–could significantly enhance the accuracy of future research on dengue transmission.

The ARIMA model is a robust time-series analysis tool that has been extensively employed for the prediction of infectious diseases [74–77]. In addition to leveraging historical time-series data, this model can account for seasonality and incorporate covariates, making it a valuable tool for forecasting and analyzing influencing factors [78]. Time-series data are often complex, and exhibit seasonality or trends that may introduce bias or spurious results in predictions. However, the ARIMA model offers the advantage of differencing the data at various lags to achieve stationarity, mitigating these challenges. Furthermore, the application of a seasonal ARIMA model allows for the explicit modelling of seasonality within the dataset, enhancing the model’s capacity to capture the cyclical patterns inherent in many infectious diseases [77]. Infectious diseases frequently exhibit non-stationary characteristics and cointegration, which can lead to spurious regression results that are often misleading and lack interpretability [56]. In our analysis, we observed seasonality and trends in the dengue dataset on both daily and monthly scales across different periods. This was also evident in the climatic variables, where dependent and independent variables showed associations extending beyond a single period. The ARDL model presents key advantages in these contexts, as it effectively addresses cointegration and demonstrates resilience against the misspecification of integration orders for the associated variables [79].

Multi-step modelling approaches, such as Long Short-Term Memory models, have previously been employed for dengue forecasting. However, to date, the association between dengue incidence and human movement using correlative approaches has not been fully established. Previous research has primarily focused on spatial and temporal analyses at smaller geographic scales, such as the Negombo region in Sri Lanka, Hermosillo in Mexico, and Fortaleza in Brazil. These studies have utilized mechanistic or mathematical models to quantify daily human mobility, applying mathematical frameworks to explore the relationship between human movement patterns and dengue transmission [35, 80, 81]. The strength of our study remains in the application of multiple modelling techniques to elucidate the complex interplay between human movement and dengue incidence. To our knowledge, this is the first study to employ multi-step correlative models that integrate climate data and consider festival events to better understand the transmission dynamics of dengue. We utilized an extensive temporal dataset spanning from January 2012 to July 2024, along with the most comprehensive spatial dataset available for Bangladesh. This allowed us to capture a substantial number of dengue cases across diverse timeframes and regions. Additionally, we rigorously evaluated model performance and goodness-of-fit metrics to compare the robustness of various models. By incorporating significant outliers, we developed a robust predictive model for the pandemic period, enabling us to estimate the number of averted cases with greater accuracy.

However, several notable limitations of this study warrant discussion. First, the spatial dengue dataset covers the period from 1st August 2019 to 31st December 2023, precluding cross-correlation analyses between the primary and secondary clusters for the year 2019. Moreover, sub-national human movement data for Bangladesh were unavailable. To address these limitations, we utilised multiple human movement indices and robust statistical analyses to provide substantial evidence. Our findings highlight the role of Dhaka district as the primary hotspot, where the absence of geographically contiguous modular communities may facilitate more rapid and widespread epidemic propagation [82]. There are also concerns related to the GCMR data used. Population dynamics, such as migration or the emergence of new employment opportunities, may alter over time, potentially influencing human movement patterns. Furthermore, Google's classification of locations may change, meaning that a similarity in values between the current time and April 2020 does not necessarily reflect identical behavior or adherence. Changes in Google’s data regarding businesses or population shifts could drive fluctuations in movement data over longer periods. Therefore, caution is advised when interpreting the outcome of this study. Similarly, the OxCGRT data for Bangladesh lacks region-specific granularity, which may fail to capture local variations in stringency measures and dengue incidence, limiting the precision of our analysis. Moreover, human movement indicators were only available for 3 years, limiting the ability to identify long-term trends, seasonal patterns, or cyclic variations in movement and dengue incidence. The collection of real-time spatiotemporal traffic data remains an unmet need in Bangladesh, which could significantly enhance future analyses.

Additionally, dengue incidence is likely influenced by a complex interplay of factors, including climate variability, vector population dynamics, public health interventions, and socioeconomic conditions—many of which were not addressed in this study [83, 84]. Certain important climatic variables that could have been relevant were not included in the ARDL model [11]. While such an analysis was beyond the scope of this research, it highlights potential avenues for future investigation. The conclusions derived from our analyses require validation in prospective studies, ideally conducted in Bangladesh or other regions with a high dengue burden, to ensure robustness. This validation should incorporate alternative methodologies that could facilitate the development of targeted preventive strategies. For instance, species distribution modelling-known for its strong predictive capabilities offers valuable insights into dengue transmission dynamics and vector habitat [85]. For the ARDL analysis, we identified a consistent fluctuation in dengue incidence occurring on Fridays, which contributed to increased residual variation and significant correlations at lag 7 and 14 in both the ACF and PACF plots of the residuals. However, analyses based on weekly or monthly aggregated data may not exhibit these weekly fluctuations. These periodic fluctuations could be attributed to several factors, including reduced hospital services, staffing levels, patient behavior, reporting mechanisms, and cultural or religious practices [86–88]. Furthermore, during the first Eid in 2024, additional contributing factors such as mosquito population growth and vectorial capacity—both of which are heavily influenced by climatic conditions—could have influenced the observed changes in dengue infection rates. We recommend the implementation of stringent vector control measures in areas used for large-scale gatherings or open-ground mass prayers, such as Gor-E-Shahid Eidgah Maidan, Sholakhia, Bishwa Ijtema, or Durga Puja celebrations, to mitigate the risk of viral transmission. Eid generates the highest levels of human movement compared to other religious and cultural festivals in Bangladesh. Additionally, the shorter duration of holidays for other festivals limits their impact. However, future studies should investigate these events to assess their potential role in dengue transmission risk.

The dengue surveillance system in Bangladesh began reporting district-level cases in mid-2019. In addition to the high number of cases reported in 2019 and 2022, the incidence in 2023 surpassed all previous records. These fluctuations highlight the need to strengthen the surveillance system for infectious diseases, ensuring accurate calculation of the actual number of cases. Currently, 77 public, private, and autonomous hospitals report dengue cases from Dhaka city, while 64 districts, including Dhaka, provide reports that are published by the Health Emergency Operation Center and Control Room under the Directorate General of Health Services, Bangladesh [39]. Considering the potential influence of regional surveillance systems on biased reporting, case notification at the administrative level 3 (Upazila level) or post office level may offer deeper insights and facilitate more effective policy implementation [44].

We were unable to incorporate mosquito population data, such as Breteau, Container, or Larval indices, due to a lack of available data. It is important to note that these indices, along with related strategic policies, are primarily based on the Dhaka metropolitan area [89]. We recommend that authorities collect a range of mosquito reproduction indices, assess the efficacy of control measures, and disseminate reproducible data. Conducting demographic health surveys, including serological testing of a representative sample and documenting historical travel destinations, could provide valuable insights into immune responses, the actual disease burden, and the formulation of health policies [90]. Furthermore, expanding serotype analysis to include a greater number of samples is essential in light of the potential threats posed by DENV genotype variability.

## Conclusions

In summary, this study employed both spatial and temporal analyses utilizing fourteen human movement-related indices, along with the Eid festival, to quantify the association with dengue incidence in one of the most dengue-endemic countries globally. Furthermore, we adopted a multi-step modelling approach to incorporate various factors, thereby enhancing the robustness of our analysis. Our findings indicate that rapid human movement is a significant factor closely associated with dengue transmission and outbreaks.

We utilized a long-term dengue database at a finer spatial resolution available in Bangladesh, which is instrumental in developing a strong early warning system. This research will aid policymakers in implementing comprehensive interventions targeting disease hotspots. By identifying the disease epicentre and its positive associations with surrounding districts, our study will contribute to predicting epidemic potential across various regions. Consequently, the identification of high-risk areas that exhibit strong correlations with the primary cluster, particularly Dhaka district, will facilitate prompt responses to mitigate the disease burden.

This preparedness will enable local authorities to effectively manage and respond to any unusual outbreaks. Additionally, this research may open avenues for investigating other vector-borne diseases such as Japanese encephalitis, malaria, and chikungunya, and could be further applied or validated in other dengue-endemic countries.

## Supplementary Information

Supplementary Material 1

## Data Availability

The datasets used and/or analysed during the current study are available from the corresponding author on reasonable request.

## Abbreviations

WHO: World Health Organization
GCMR: Google’s community mobility reports
ARIMA: Autoregressive integrated moving average
SARIMA: Seasonal autoregressive integrated moving average
ARDL: Autoregressive distributed lag
PHSM: Public health and social measures
OxCGRT: Oxford Coronavirus Government Response Tracker
SI: Stringency index
SPSS: Statistical package for the social sciences
ACF: Autocorrelation function
PACF: Partial autocorrelation function
STL: Seasonal-trend decomposition with LOESS
MA: Moving average
AR: Autoregressive
RMSE: Root mean square error
BIC: Bayesian information criterion
MAPE: Mean absolute percentage error
VAR: Vector autoregressive
RR: Relative risk
SE: Standard error
DC: Dengue case
T: Temperature
H: Humidity
GWR: Geographically weighted regression
DENV: Dengue virus
