# Supplementary material for: Rapid human movement and dengue transmission in Bangladesh: a spatial and temporal analysis based on different policy measures of COVID-19 pandemic and Eid festival
Source: Infect Dis Poverty. 2024 Dec 26;13:99. doi: 10.1186/s40249-024-01267-4 (PMC11670399; doi:10.1186/s40249-024-01267-4)
Supplement: Supplementary file 1 — Supplementary Material 1 [file 40249_2024_1267_MOESM1_ESM.docx]

**Supplementary Material**

**Supplement to:** Jahirul Islam^1^, Wenbiao Hu^1^.

Rapid human movement and dengue transmission in Bangladesh: A spatial and temporal analysis based on different policy measures of COVID-19 pandemic and Eid festival

^1^ Ecosystem Change and Population Health Research Group, Centre for Immunology and Infection Control, School of Public Health and Social Work, Queensland University of Technology, Brisbane, Australia

**Research question:** The aim of this research is to conduct a spatial and temporal analysis of dengue incidence in Bangladesh by using robust spatial and time-series models to explore the significant spatial clusters, disease epicentre, influence of EID festivals, and investigate the association of monthly incidence with fourteen human movement related indices.

**Table of Contents**

[**Fig. S1.** Stringency sub-index over time: Impact on eight public health and social measures (January 2020–December 2022). 6](#_Toc183641442)

[**Fig S2.** Google’s community mobility sub-indices over time: Impact on six measures (15 February 2020–15 October 2022). 10](#_Toc183641443)

[**Fig. S3.** Autocorrelation function and partial autocorrelation function plots for seasonal autoregressive integrated moving average model (2012–2019) using expert modeler. 11](#_Toc183641444)

[**Fig. S4.** Autocorrelation function and partial autocorrelation function plots for seasonal autoregressive integrated moving average model (2012–2019) of model 2. 12](#_Toc183641445)

[**Fig. S5.** Autocorrelation function and partial autocorrelation function plots for seasonal autoregressive integrated moving average model (2012–2019) of model 3. 12](#_Toc183641446)

[**Fig. S6.** Autocorrelation function and partial autocorrelation function plots for seasonal autoregressive integrated moving average model (2012–2019) of model 4. 13](#_Toc183641447)

[**Fig. S7.** Cross-correlation between Dhaka and all other districts ranged from 2019–2023. Three-time periods were used where the before-pandemic data is the yearly aggregated data for 2019, during pandemic period represents the aggregated number of incidences from 2020–22, and the post-pandemic period data included the yearly number of cases found in 2023. 16](#_Toc183641448)

[**Fig. S8.** Autocorrelation function and partial autocorrelation function plots for seasonal autoregressive integrated moving average model 1 configurations. 17](#_Toc183641449)

[**Fig. S9.** Autocorrelation function and partial autocorrelation function plots for seasonal autoregressive integrated moving average model 2 configurations. 17](#_Toc183641450)

[**Fig. S10.** Autocorrelation function and partial autocorrelation function plots for seasonal autoregressive integrated moving average model 3 configurations. 18](#_Toc183641451)

[**Fig. S11.** Autocorrelation function and partial autocorrelation function plots for seasonal autoregressive integrated moving average model 4 configurations. 19](#_Toc183641452)

[**Fig. S12.** Autocorrelation function and partial autocorrelation function plots for seasonal autoregressive integrated moving average model 5 configurations. 19](#_Toc183641453)

[**Fig. S13.** Histogram of residual for autoregressive integrated moving average forecast model of Eid 1, 2023. 20](#_Toc183641454)

[**Fig. S14.** Histogram of residual for autoregressive integrated moving average forecast model of Eid 2, 2023. 21](#_Toc183641455)

[**Fig. S15.** Histogram of residual for autoregressive integrated moving average forecast model of Eid 1, 2024. 22](#_Toc183641456)

[**Fig. S16.** Residuals, autocorrelation function, and partial autocorrelation function plots for the autoregressive distributed lag model of Eid 1, 2023. 25](#_Toc183641457)

[**Fig. S17.** Residuals, autocorrelation function, and partial autocorrelation function plots for the autoregressive distributed lag model of Eid 2, 2023. 26](#_Toc183641458)

[**Fig. S18.** Residuals, autocorrelation function, and partial autocorrelation function plots for the autoregressive distributed lag model of Eid 1, 2024. 27](#_Toc183641459)

[**Table S1** 11](#_Toc183641460)

[**Table S2** 11](#_Toc183641461)

[**Table S3** 13](#_Toc183641462)

[**Table S4** 14](#_Toc183641463)

[**Table S5** 23](#_Toc183641464)

**Stringency index (SI)**

OxCGRT collects publicly available information on indicators of government response. These indicators take policies such as school closures, travel bans, etc. and record them on an ordinal scale. The remainder is financial indicators, such as fiscal or monetary measures. OxCGRT measures the variation in governments’ responses using its COVID-19 Government Response Stringency Index. This composite measure is a simple additive score of nine indicators measured on an ordinal scale, rescaled to vary from 0–100. Please note that this measure is for comparative purposes only and should not be interpreted as a rating of the appropriateness or effectiveness of a country's response. It also includes a 'COVID-19 Containment and Health Response' index which is based on the metrics used in the 'Stringency Index' plus testing policy, contact tracing, face coverings and vaccine policy.

The specific policy and response categories are coded as follows:

School closures: 0 - No measures 1 - recommend closing 2 - Require closing (only some levels or categories, e.g. just high school, or just public schools) 3 - Require closing all levels No data - blank

Workplace closures: 0 - No measures 1 - recommend closing (or work from home) 2 - require closing (or work from home) for some sectors or categories of workers 3 - require closing (or work from home) all but essential workplaces (e.g. grocery stores, doctors) No data - blank

Cancel public events: 0- No measures 1 - Recommend cancelling 2 - Require cancelling No data - blank

Restrictions on gatherings: 0 - No restrictions 1 - Restrictions on very large gatherings (the limit is above 1,000 people) 2 - Restrictions on gatherings between 100-1,000 people 3 - Restrictions on gatherings between 10-100 people 4 - Restrictions on gatherings of less than 10 people No data - blank

Close public transport: 0 - No measures 1 - Recommend closing (or significantly reduce volume/route/means of transport available) 2 - Require closing (or prohibit most citizens from using it)

Stay at home: 0 - No measures 1 - recommend not leaving house 2 - require not leaving house with exceptions for daily exercise, grocery shopping, and ‘essential’ trips 3 - Require not leaving house with minimal exceptions (e.g., allowed to leave only once every few days, or only one person can leave at a time, etc.) No data - blank

Restrictions on internal movement: 0 - No measures 1 - Recommend movement restriction 2 - Restrict movement

International travel controls: 0 - No measures 1 - Screening 2 - Quarantine arrivals from high-risk regions 3 - Ban on high-risk regions 4 - Total border closure No data - blank

The following link provides necessary information regarding how each stringency index was calculated: <https://github.com/OxCGRT/covid-policy-tracker/blob/master/documentation/index_methodology.md>

**
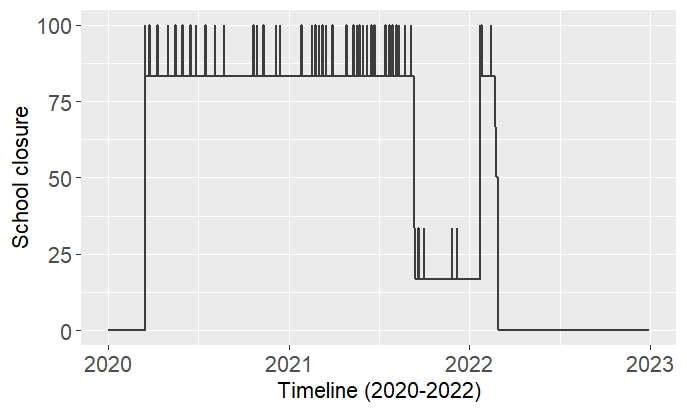
**

**
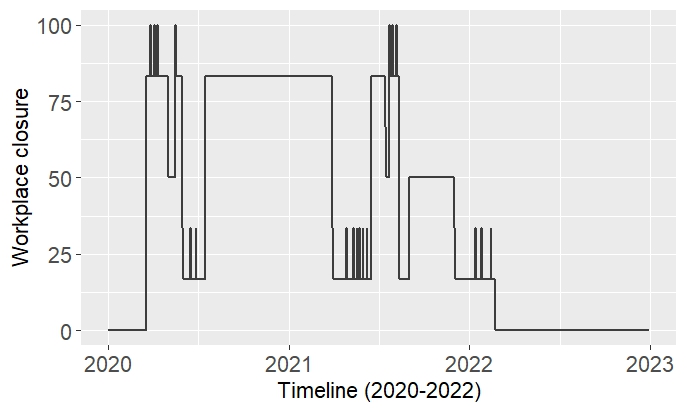
**

**
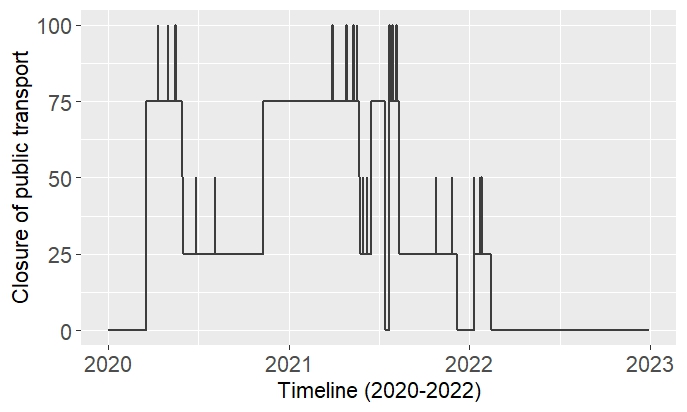
**

**
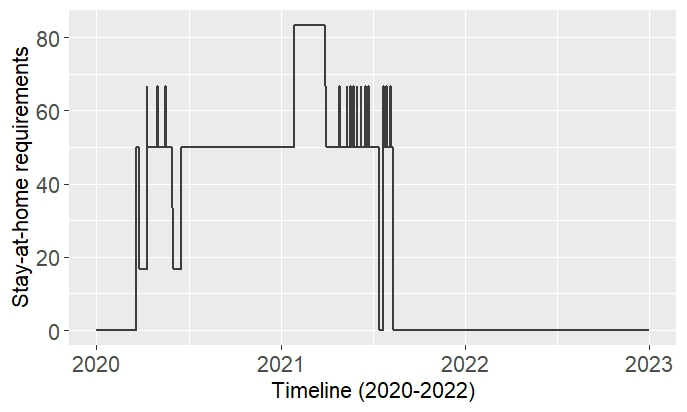
**

**
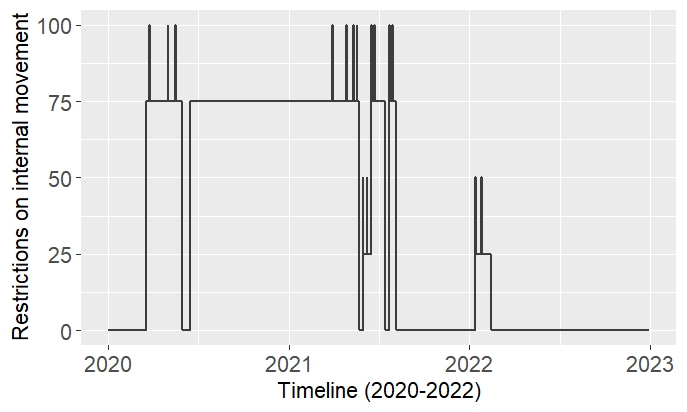
**

**
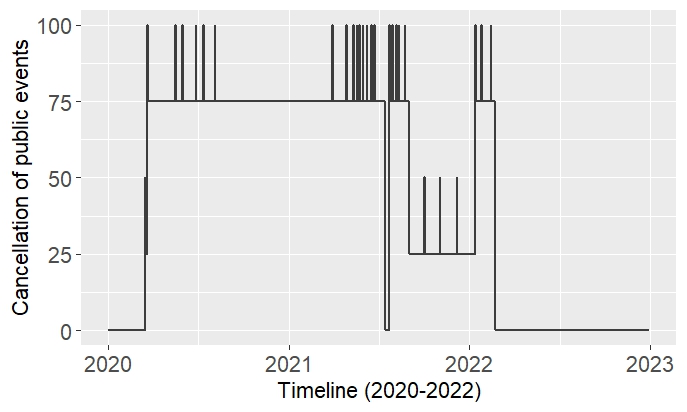
**

**
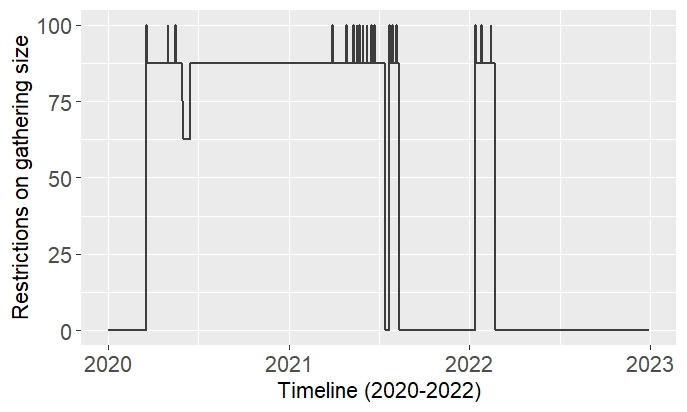

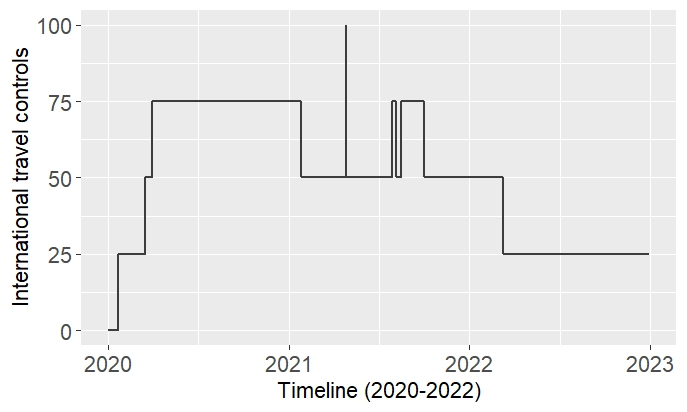

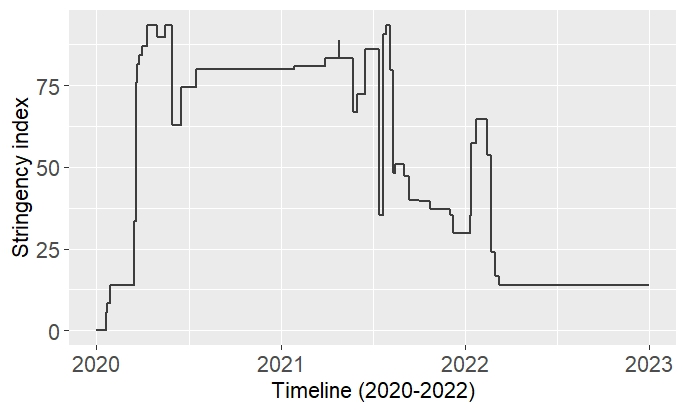
**

**Fig. S1.** Stringency sub-index over time: Impact on eight public health and social measures (January 2020–December 2022).

**Google Community Mobility Reports (GCMR)**

Data available at: <https://www.google.com/covid19/mobility/>

In order to ensure the usefulness of the reports, GCMR employs categories to classify certain locations that share comparable attributes, with the aim of providing advice on social distancing measures. As an illustration, GCMR merge the supermarket and pharmacy categories since they are typically regarded as necessary excursions.

Every major category comprises numerous distinct sorts of locations, some of which may not be immediately apparent. The table below displays a limited selection of locations encompassed within two illustrative categories:

| **Parks** | **Transit stations** |
| --- | --- |
| Public garden | Subway station |
| Castle | Sea port |
| National forest | Taxi stand |
| Camp ground | Highway rest stop |
| Observation deck | Car rental agency |


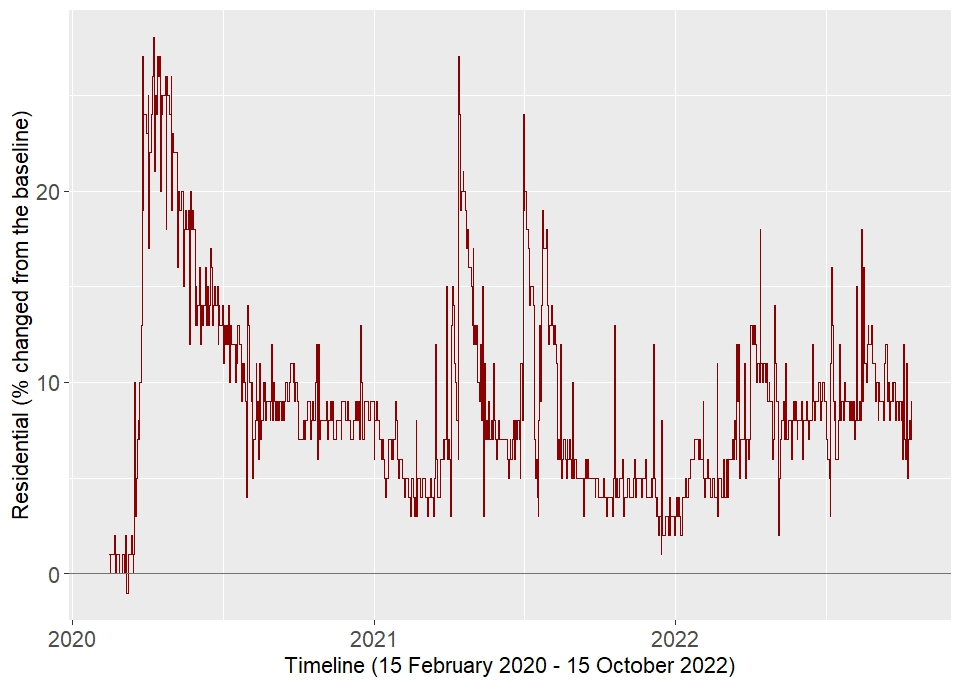


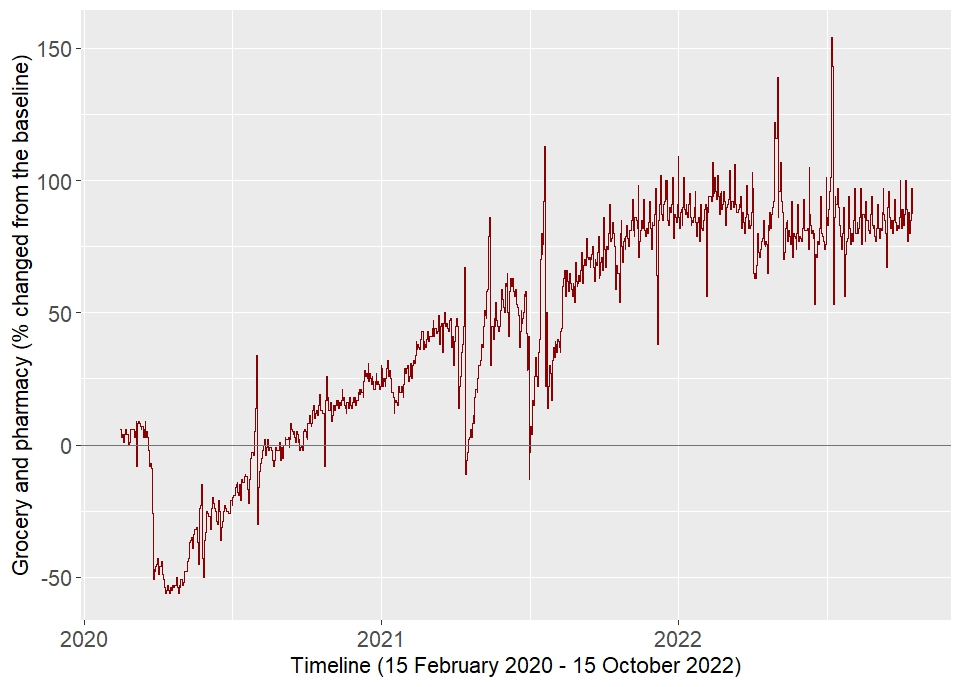


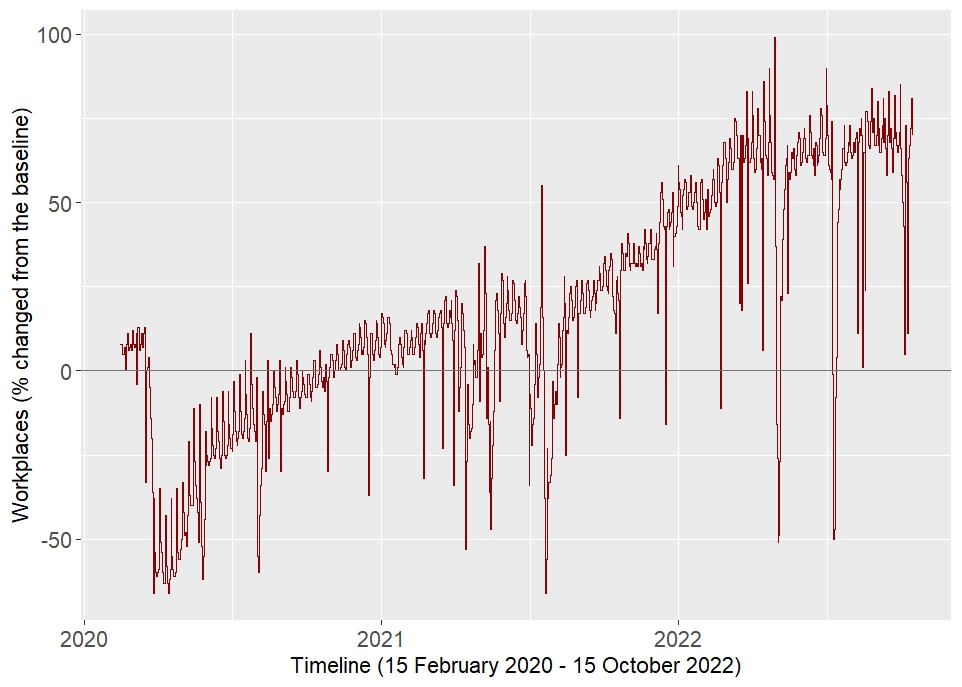


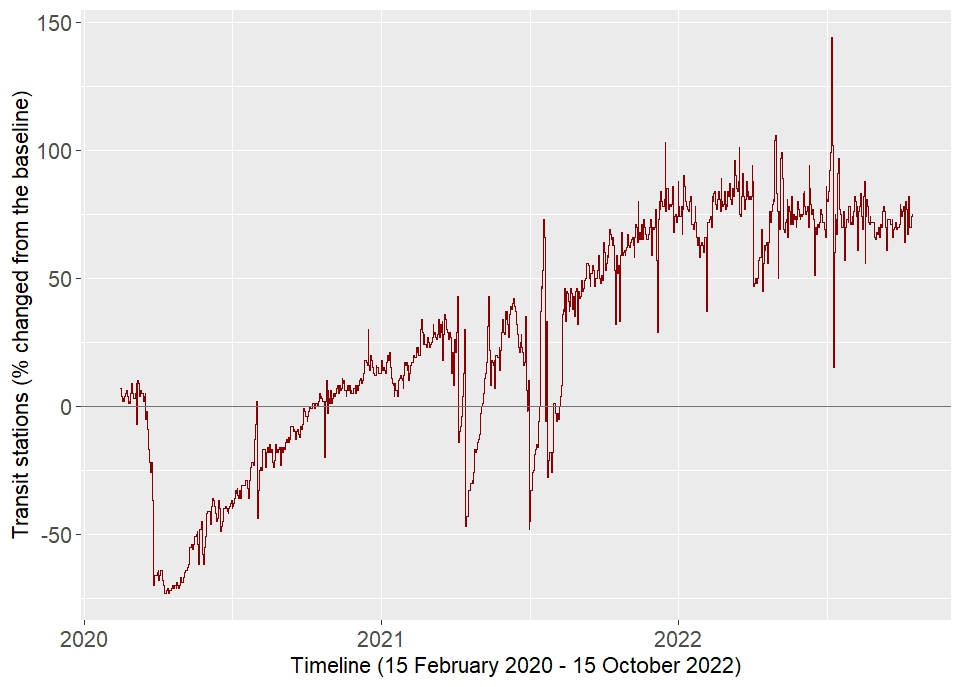


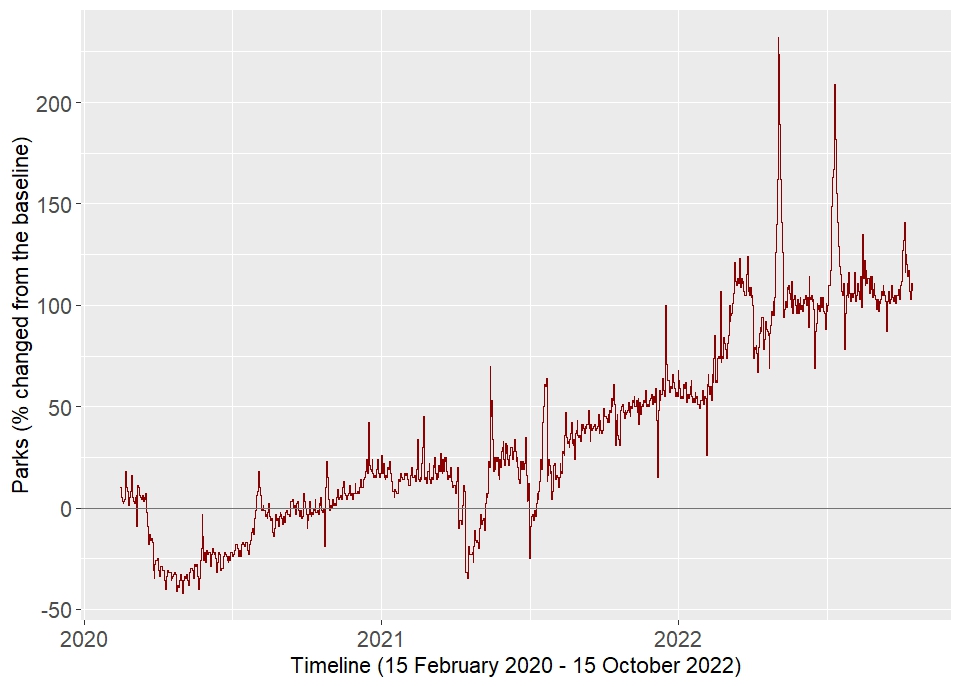


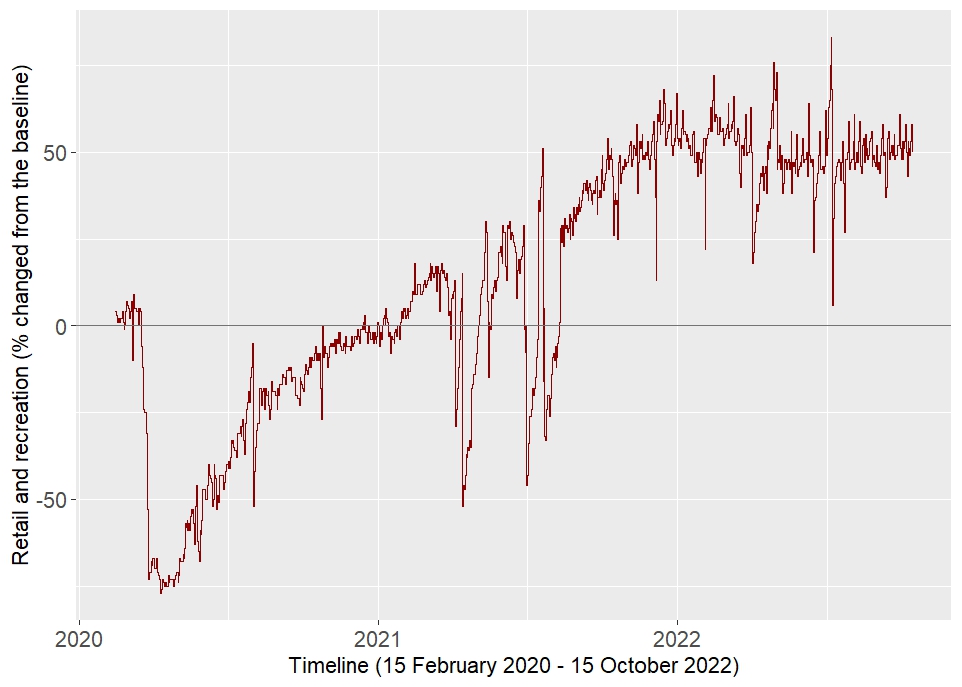


**Fig S2.** Google’s community mobility sub-indices over time: Impact on six measures (15 February 2020–15 October 2022).

More information: <https://support.google.com/covid19-mobility/answer/9824897?hl=en&ref_topic=9822927&sjid=2068215645170879558-AP>

**Table S1**

Evaluation metrics for various SARIMA model configurations.

| Model | ${(p,d,q)\times(P,D,Q)}_{12}$ | BIC | MAPE | RMSE | *R^2^* |
| --- | --- | --- | --- | --- | --- |
| 1 | (2,0,1) $\boldsymbol{\times}$ (0,1,0)_12_ | 5.96 | 97.94 | 14.57 | .89 |
| 2 | (2,0,0) $\boldsymbol{\times}$ (0,1,0)_12_ | 6.09 | 102.93 | 16.77 | .85 |
| 3 | (2,1,0) $\boldsymbol{\times}$ (0,1,0)_12_ | 5.99 | 57.91 | 15.85 | .87 |
| 4 | (0,1,2) $\boldsymbol{\times}$ (0,1,0)_12_ | 5.87 | 62.36 | 14.90 | .88 |
| 5 | Expert modeler (0,2,0) | 5.25 | 48.07 | 13.06 | .87 |
| *BIC* Bayesian information criterion; MAPE: mean absolute percentage error; *RMSE* root mean square error. | | | | | |

**Table S2**

seasonal autoregressive integrated moving average model comparison using the 2012-2019 data.

| ${(p,d,q)\times(P,D,Q)}_{12}$ | BIC | MAPE | RMSE | *R^2^* |
| --- | --- | --- | --- | --- |
| Model 1: Expert modeler |  |  |  |  |
| (0,1,0) $\boldsymbol{\times}$ (0,1,0)_12_ | 3.08 | 25.91 | 3.67 | .99 |
| Model 2 | | | | |
| (2,1,0) $\boldsymbol{\times}$ (0,1,0)_12_ | 5.99 | 33.68 | 18.44 | .67 |
| Model 3 | | | | |
| (2,1,1) $\boldsymbol{\times}$ (0,1,0)_12_ | 5.89 | 45.35 | 17.16 | .74 |
| Model 4 | | | | |
| (0,1,2) $\boldsymbol{\times}$ (0,1,0)_12_ | 5.91 | 49.18 | 17.78 | .72 |
| BIC Bayesian information criterion; MAPE: mean absolute percentage error; RMSE root mean square error.  The dengue data used here were monthly aggregated data. | | | | |


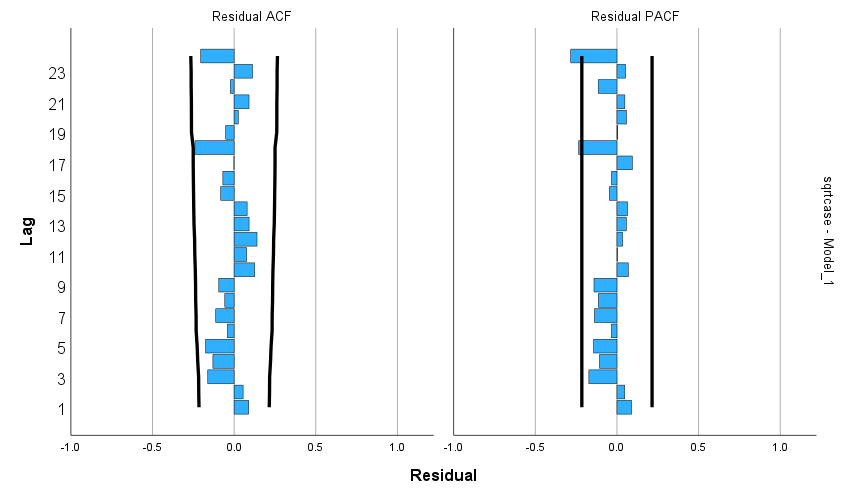


**Fig. S3.** Autocorrelation function and partial autocorrelation function plots for seasonal autoregressive integrated moving average model (2012–2019) using expert modeler.


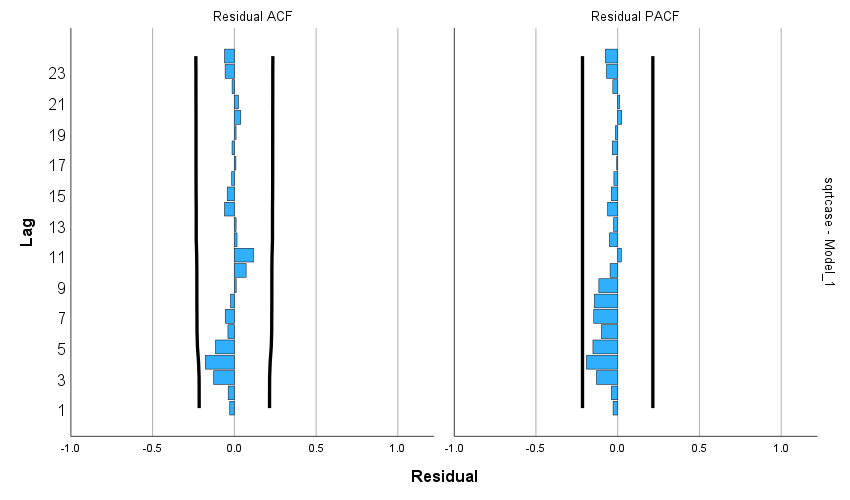


**Fig. S4.** Autocorrelation function and partial autocorrelation function plots for seasonal autoregressive integrated moving average model (2012–2019) of model 2.


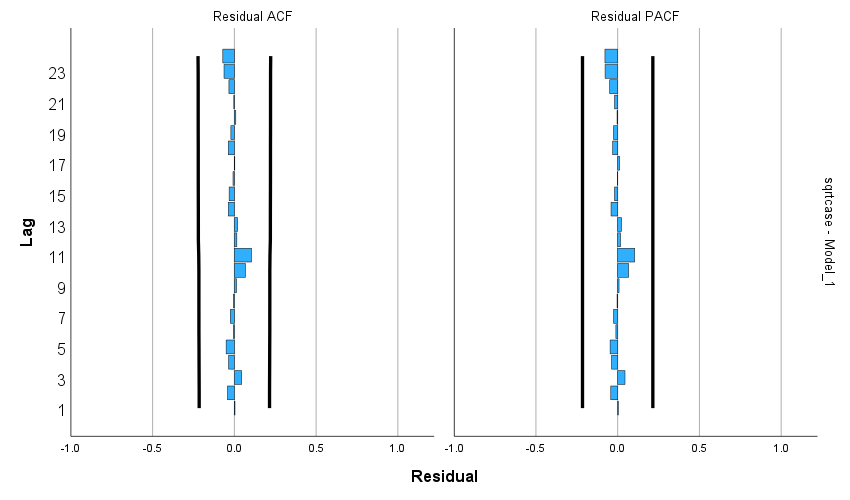


**Fig. S5.** Autocorrelation function and partial autocorrelation function plots for seasonal autoregressive integrated moving average model (2012–2019) of model 3.


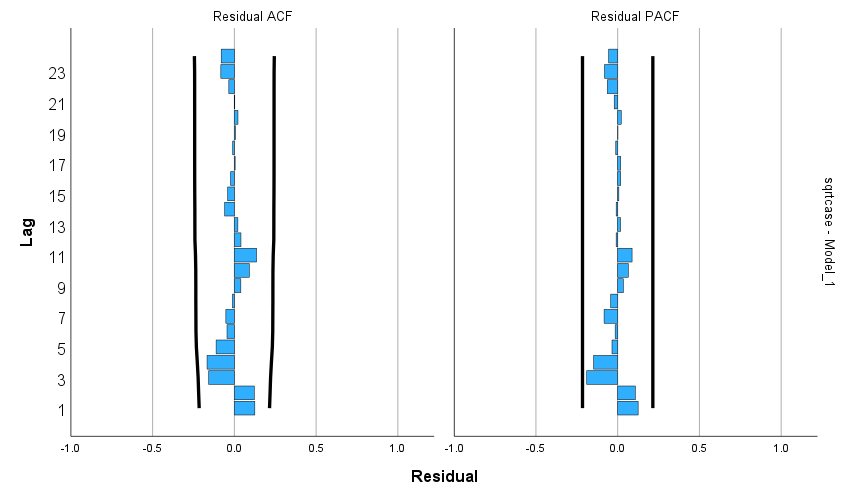


**Fig. S6.** Autocorrelation function and partial autocorrelation function plots for seasonal autoregressive integrated moving average model (2012–2019) of model 4.

**Table S3**

Augmented dickey-fuller test test for three Eid periods daily data

| Period | Variables | ADF | | | |
| --- | --- | --- | --- | --- | --- |
|  |  | Level | *P*-value | First difference | *P*-value |
| Eid 1 (2023) | Dengue case | -1.40 | 0.82 | -6.83 | 0.01 |
|  | Temperature | -2.66 | 0.30 | -3.02 | 0.15 |
|  | Humidity | -3.20 | 0.09 | -5.16 | 0.01 |
|  | Precipitation | -3.23 | 0.08 | -6.00 | 0.01 |
| Eid 2 (2023) | Dengue case | 1.62 | 0.99 | -4.51 | 0.01 |
|  | Temperature | -3.22 | 0.08 | -4.94 | 0.01 |
|  | Humidity | -2.12 | 0.52 | -5.33 | 0.01 |
|  | Precipitation | -3.73 | 0.02 | -4.72 | 0.01 |
| Eid 1 (2024) | Dengue case | -3.68 | 0.03 | -7.81 | 0.01 |
|  | Temperature | -2.53 | 0.35 | -3.49 | 0.05 |
|  | Humidity | -2.58 | 0.33 | -4.87 | 0.01 |
|  | Precipitation | -3.29 | 0.07 | -5.83 | 0.01 |
| *ADF* augmented dickey-fuller test. | | | | | |

**Table S4**

Vector autoregressive Model Selection Criteria Results.

| Selection | Criteria | 1 | 2 | 3 | 4 | 5 | 6 | 7 | 8 | 9 | 10 |
| --- | --- | --- | --- | --- | --- | --- | --- | --- | --- | --- | --- |
| Eid 1: 2023 | | | | | | | | | | | |
| Dengue |  |  |  |  |  |  |  |  |  |  |  |
| 6 | AIC^a^ (n) | 4.30 | 4.22 | 4.20 | 4.17 | 4.18 | 3.79 | 3.80 | 3.81 | 3.81 | 3.79 |
| 6 | HQ^b^ (n) | 4.32 | 4.26 | 4.25 | 4.23 | 4.25 | 3.87 | 3.90 | 3.92 | 3.93 | 3.93 |
| 6 | SC^c^ (n) | 4.36 | 4.32 | 4.33 | 4.33 | 4.36 | 4.00 | 4.05 | 4.08 | 4.11 | 4.13 |
| 6 | FPE^d^ (n) | 73.46 | 68.26 | 66.88 | 64.94 | 65.09 | 44.06 | 44.91 | 45.19 | 45.16 | 44.46 |
| Temperature |  |  |  |  |  |  |  |  |  |  |  |
| 2 | AIC (n) | -0.46 | -0.47 | -0.45 | -0.42 | -0.40 | -0.42 | -0.40 | -0.38 | -0.37 | -0.36 |
| 1 | HQ (n) | -0.43 | -0.43 | -0.40 | -0.36 | -0.32 | -0.34 | -0.30 | -0.27 | -0.25 | -0.22 |
| 1 | SC (n) | -0.40 | -0.37 | -0.32 | -0.27 | -0.21 | -0.21 | -0.15 | -0.10 | -0.07 | -0.02 |
| 2 | FPE (n) | 0.63 | 0.63 | 0.64 | 0.66 | 0.67 | 0.66 | 0.67 | 0.69 | 0.69 | 0.70 |
| Humidity |  |  |  |  |  |  |  |  |  |  |  |
| 1 | AIC (n) | 3.21 | 3.22 | 3.23 | 3.25 | 3.27 | 3.27 | 3.29 | 3.31 | 3.33 | 3.33 |
| 1 | HQ (n) | 3.23 | 3.25 | 3.28 | 3.31 | 3.34 | 3.35 | 3.39 | 3.42 | 3.45 | 3.47 |
| 1 | SC (n) | 3.27 | 3.31 | 3.35 | 3.40 | 3.45 | 3.48 | 3.53 | 3.59 | 3.64 | 3.67 |
| 1 | FPE (n) | 24.76 | 24.94 | 25.26 | 25.68 | 26.33 | 26.27 | 26.90 | 27.50 | 28.02 | 28.11 |
| Precipitation |  |  |  |  |  |  |  |  |  |  |  |
| 4 | AIC (n) | 3.39 | 3.30 | 3.30 | 3.27 | 3.29 | 3.30 | 3.32 | 3.32 | 3.34 | 3.36 |
| 4 | HQ (n) | 3.41 | 3.34 | 3.35 | 3.33 | 3.36 | 3.38 | 3.41 | 3.43 | 3.46 | 3.49 |
| 2 | SC (n) | 3.45 | 3.39 | 3.42 | 3.42 | 3.47 | 3.51 | 3.56 | 3.60 | 3.64 | 3.69 |
| 4 | FPE (n) | 29.61 | 27.10 | 27.05 | 26.38 | 26.81 | 27.12 | 27.59 | 27.81 | 3.34 | 3.36 |
| Eid 2: 2023 | | | | | | | | | | | |
| Dengue |  |  |  |  |  |  |  |  |  |  |  |
| 10 | AIC (n) | 11.09 | 11.04 | 11.05 | 11.07 | 11.09 | 10.79 | 10.22 | 9.90 | 9.89 | 9.89 |
| 8 | HQ (n) | 11.12 | 11.08 | 11.10 | 11.13 | 11.16 | 10.87 | 10.32 | 10.01 | 10.01 | 10.02 |
| 8 | SC (n) | 11.16 | 11.13 | 11.17 | 11.22 | 11.27 | 11.00 | 10.46 | 10.17 | 10.19 | 10.22 |
| 10 | FPE (n) | 65829.38 | 62500.33 | 63011.87 | 64049.55 | 65272.70 | 48460.91 | 27403.94 | 19926.41 | 19709.16 | 19691.28 |
| Temperature |  |  |  |  |  |  |  |  |  |  |  |
| 2 | AIC (n) | 0.23 | 0.21 | 0.24 | 0.23 | 0.24 | 0.26 | 0.28 | 0.28 | 0.30 | 0.33 |
| 2 | HQ (n) | 0.25 | 0.25 | 0.28 | 0.29 | 0.31 | 0.34 | 0.37 | 0.39 | 0.43 | 0.46 |
| 1 | SC (n) | 0.29 | 0.30 | 0.36 | 0.38 | 0.42 | 0.47 | 0.52 | 0.55 | 0.61 | 0.66 |
| 2 | FPE (n) | 1.25 | 1.23 | 1.27 | 1.25 | 1.27 | 1.29 | 1.32 | 1.32 | 1.36 | 1.39 |
| ^a^*AIC* Akaike information criterion; ^b^ *HQ* Hannan-Quinn information criterion; ^c^ *SC* schwarz criterion or Bayesian information criterion; ^d^ *FPE* final prediction error. | | | | | | | | | | | |

**Table S4** (Continued.)

| Selection | Criteria | 1 | 2 | 3 | 4 | 5 | 6 | 7 | 8 | 9 | 10 |
| --- | --- | --- | --- | --- | --- | --- | --- | --- | --- | --- | --- |
| Humidity |  |  |  |  |  |  |  |  |  |  |  |
| 1 | AIC (n) | 3.35 | 3.37 | 3.38 | 3.40 | 3.40 | 3.42 | 3.43 | 3.40 | 3.42 | 3.45 |
| 1 | HQ (n) | 3.37 | 3.41 | 3.43 | 3.46 | 3.47 | 3.50 | 3.53 | 3.51 | 3.54 | 3.58 |
| 1 | SC (n) | 3.41 | 3.46 | 3.50 | 3.55 | 3.58 | 3.63 | 3.68 | 3.68 | 3.72 | 3.78 |
| 1 | FPE (n) | 28.42 | 29.14 | 29.45 | 29.94 | 29.85 | 30.48 | 31.02 | 30.05 | 30.63 | 31.45 |
| Precipitation |  |  |  |  |  |  |  |  |  |  |  |
| 8 | AIC (n) | 4.52 | 4.54 | 4.52 | 4.53 | 4.55 | 4.57 | 4.54 | 4.47 | 4.50 | 4.52 |
| 1 | HQ (n) | 4.55 | 4.57 | 4.56 | 4.59 | 4.63 | 4.65 | 4.63 | 4.58 | 4.62 | 4.65 |
| 1 | SC (n) | 4.58 | 4.63 | 4.64 | 4.68 | 4.73 | 4.78 | 4.78 | 4.74 | 4.80 | 4.85 |
| 8 | FPE (n) | 92.10 | 93.25 | 91.40 | 92.96 | 94.95 | 96.13 | 93.38 | 87.48 | 89.78 | 92.11 |
| Eid 1: 2024 | | | | | | | | | | | |
| Dengue |  |  |  |  |  |  |  |  |  |  |  |
| 6 | AIC (n) | 4.42 | 4.36 | 4.34 | 4.34 | 4.23 | 4.17 | 4.19 | 4.20 | 4.19 | 4.20 |
| 6 | HQ (n) | 4.45 | 4.40 | 4.39 | 4.40 | 4.31 | 4.26 | 4.29 | 4.31 | 4.31 | 4.34 |
| 6 | SC (n) | 4.48 | 4.45 | 4.46 | 4.50 | 4.41 | 4.38 | 4.44 | 4.48 | 4.49 | 4.54 |
| 6 | FPE (n) | 83.31 | 78.50 | 76.47 | 77.01 | 68.89 | 64.77 | 66.23 | 67.04 | 66.12 | 66.93 |
| Temperature |  |  |  |  |  |  |  |  |  |  |  |
| 1 | AIC (n) | 0.63 | 0.65 | 0.68 | 0.70 | 0.72 | 0.72 | 0.75 | 0.75 | 0.78 | 0.80 |
| 1 | HQ (n) | 0.66 | 0.69 | 0.72 | 0.76 | 0.79 | 0.81 | 0.85 | 0.86 | 0.90 | 0.94 |
| 1 | SC (n) | 0.69 | 0.75 | 0.80 | 0.85 | 0.90 | 0.94 | 0.99 | 1.03 | 1.08 | 1.14 |
| 1 | FPE (n) | 1.88 | 1.92 | 1.96 | 2.01 | 2.05 | 2.06 | 2.12 | 2.13 | 2.18 | 2.24 |
| Humidity |  |  |  |  |  |  |  |  |  |  |  |
| 1 | AIC (n) | 3.81 | 3.83 | 3.84 | 3.85 | 3.88 | 3.90 | 3.91 | 3.93 | 3.96 | 3.98 |
| 1 | HQ (n) | 3.83 | 3.87 | 3.88 | 3.91 | 3.95 | 3.99 | 4.01 | 4.04 | 4.08 | 4.11 |
| 1 | SC (n) | 3.87 | 3.92 | 3.96 | 4.00 | 4.06 | 4.11 | 4.15 | 4.21 | 4.26 | 4.31 |
| 1 | FPE (n) | 45.09 | 46.00 | 46.31 | 47.10 | 48.26 | 49.49 | 49.88 | 51.20 | 52.56 | 53.42 |
| Precipitation |  |  |  |  |  |  |  |  |  |  |  |
| 3 | AIC (n) | 3.82 | 3.71 | 3.66 | 3.67 | 3.69 | 3.71 | 3.73 | 3.75 | 3.77 | 3.79 |
| 3 | HQ (n) | 3.84 | 3.74 | 3.71 | 3.73 | 3.77 | 3.80 | 3.83 | 3.86 | 3.90 | 3.93 |
| 3 | SC (n) | 3.88 | 3.80 | 3.78 | 3.83 | 3.88 | 3.93 | 3.98 | 4.03 | 4.08 | 4.13 |
| 3 | FPE (n) | 45.58 | 40.79 | 38.85 | 39.41 | 40.24 | 41.07 | 41.90 | 42.72 | 43.64 | 44.56 |
| ^a^*AIC* Akaike information criterion; ^b^ *HQ* Hannan-Quinn information criterion; ^c^ *SC* schwarz criterion or Bayesian information criterion; ^d^ *FPE* final prediction error. | | | | | | | | | | | |


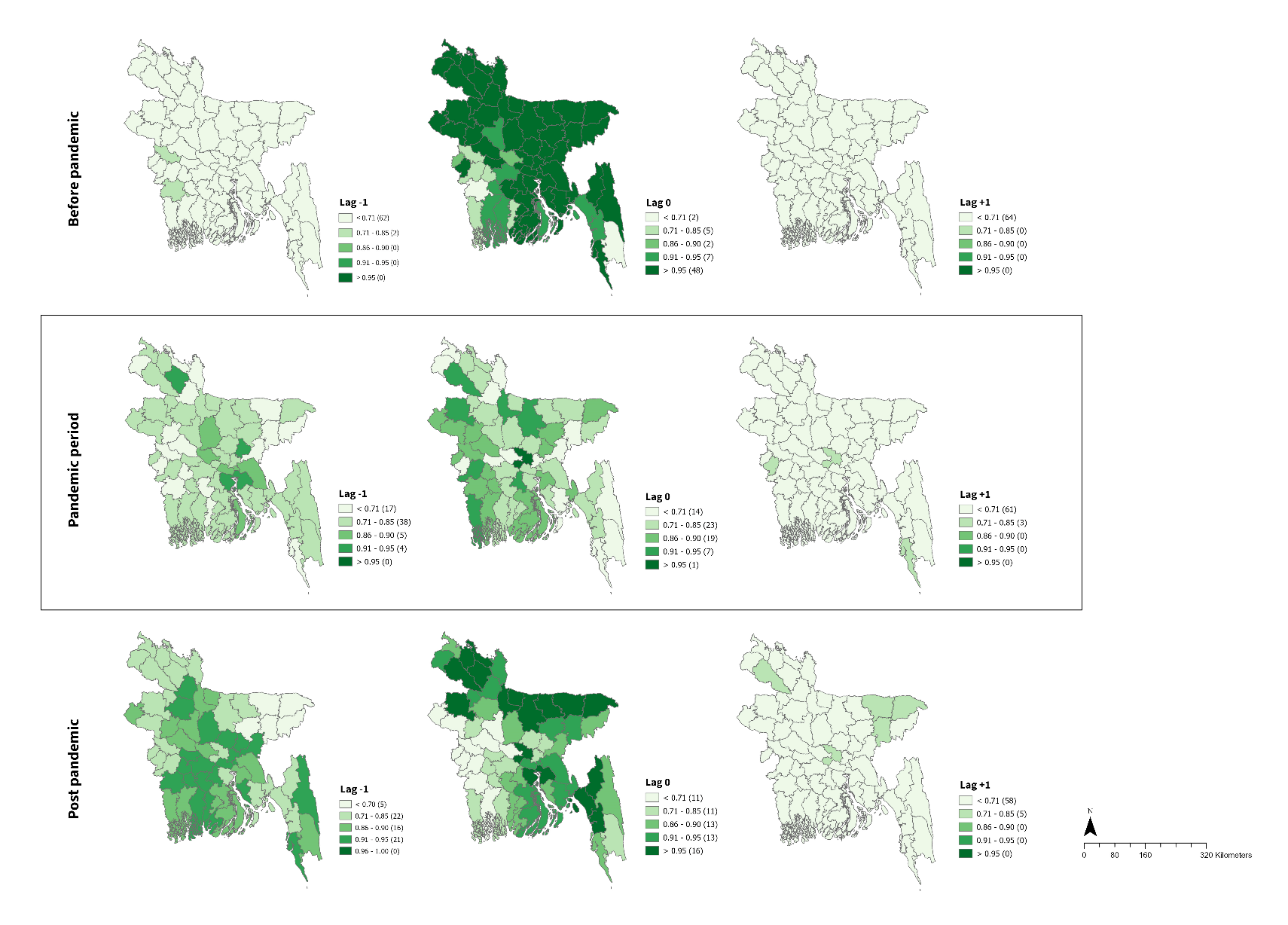


**Fig. S7.** Cross-correlation between Dhaka and all other districts ranged from 2019–2023. Three-time periods were used where the before-pandemic data is the yearly aggregated data for 2019, during pandemic period represents the aggregated number of incidences from 2020–22, and the post-pandemic period data included the yearly number of cases found in 2023.


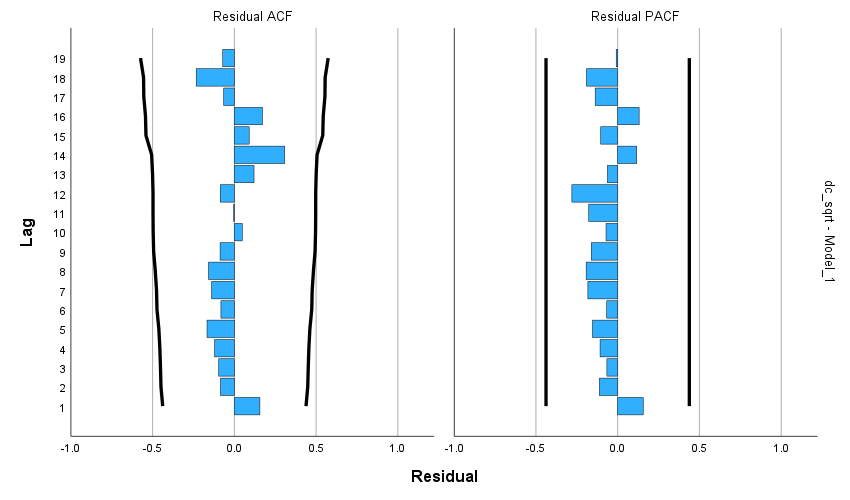


**Fig. S8.** Autocorrelation function and partial autocorrelation function plots for seasonal autoregressive integrated moving average model 1 configurations.


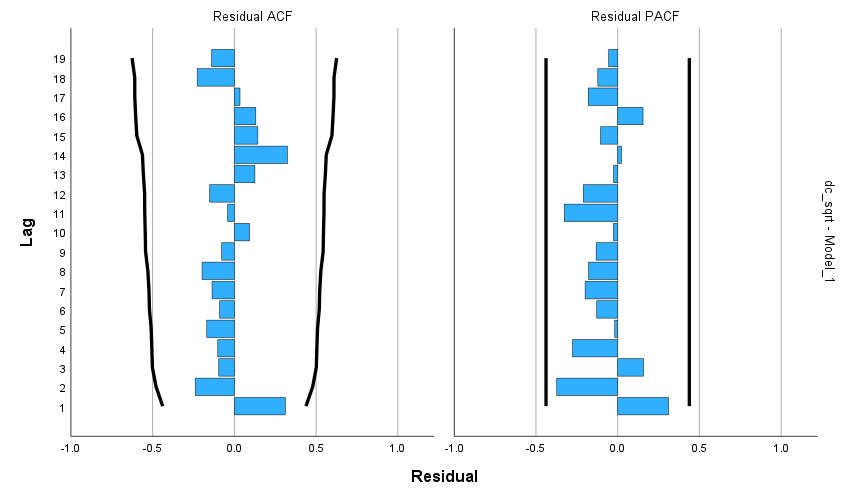


**Fig. S9.** Autocorrelation function and partial autocorrelation function plots for seasonal autoregressive integrated moving average model 2 configurations.


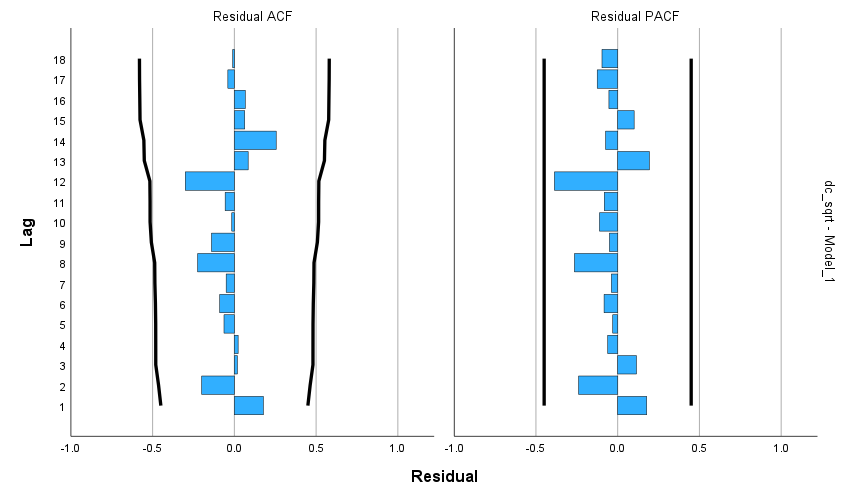


**Fig. S10.** Autocorrelation function and partial autocorrelation function plots for seasonal autoregressive integrated moving average model 3 configurations.


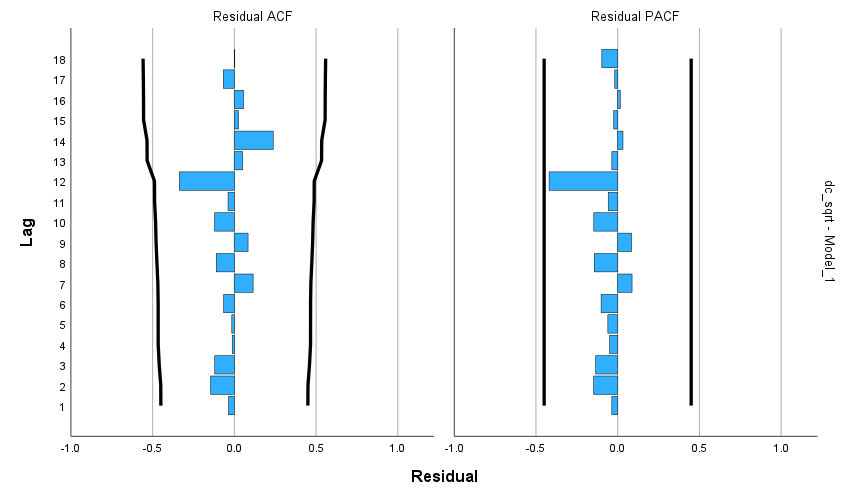


**Fig. S11.** Autocorrelation function and partial autocorrelation function plots for seasonal autoregressive integrated moving average model 4 configurations.


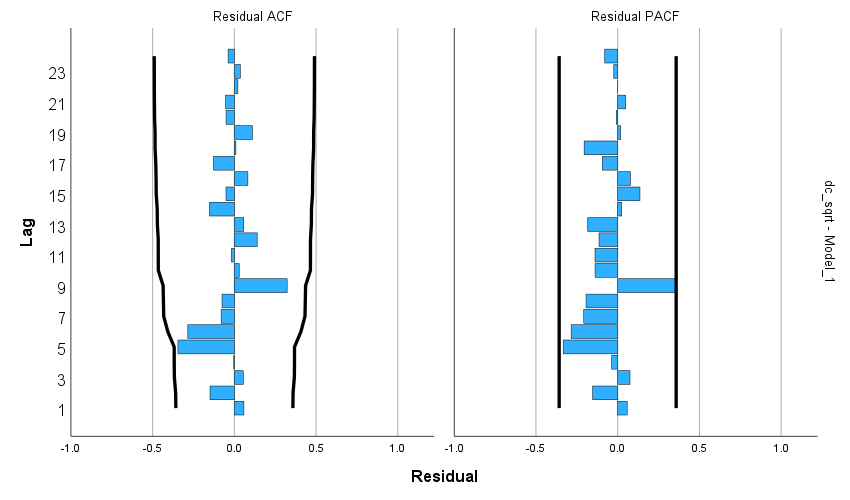


**Fig. S12.** Autocorrelation function and partial autocorrelation function plots for seasonal autoregressive integrated moving average model 5 configurations.


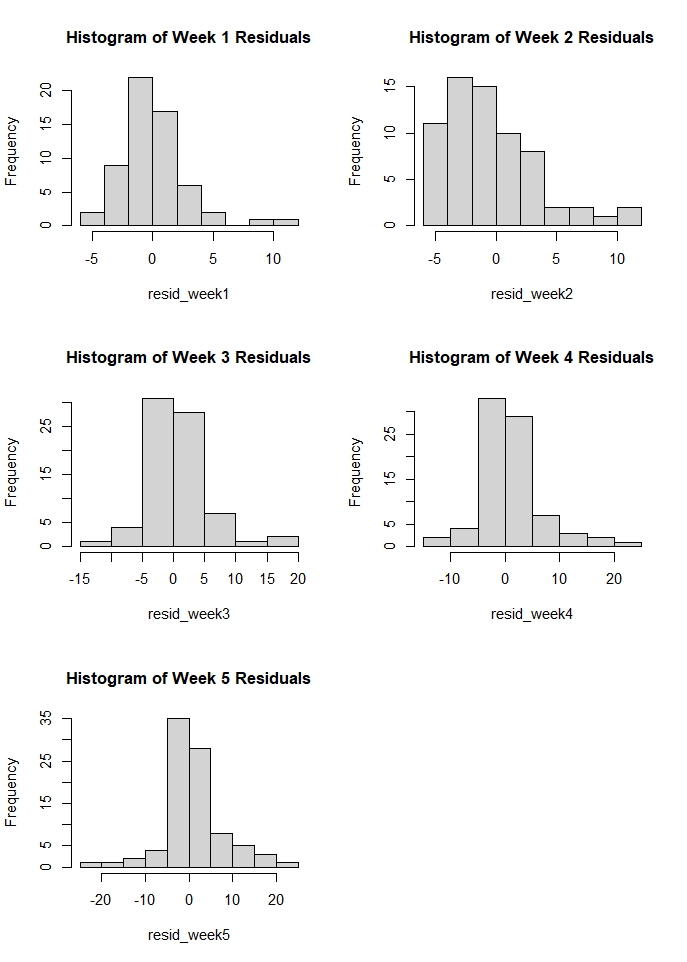


**Fig. S13.** Histogram of residual for autoregressive integrated moving average forecast model of Eid 1, 2023.


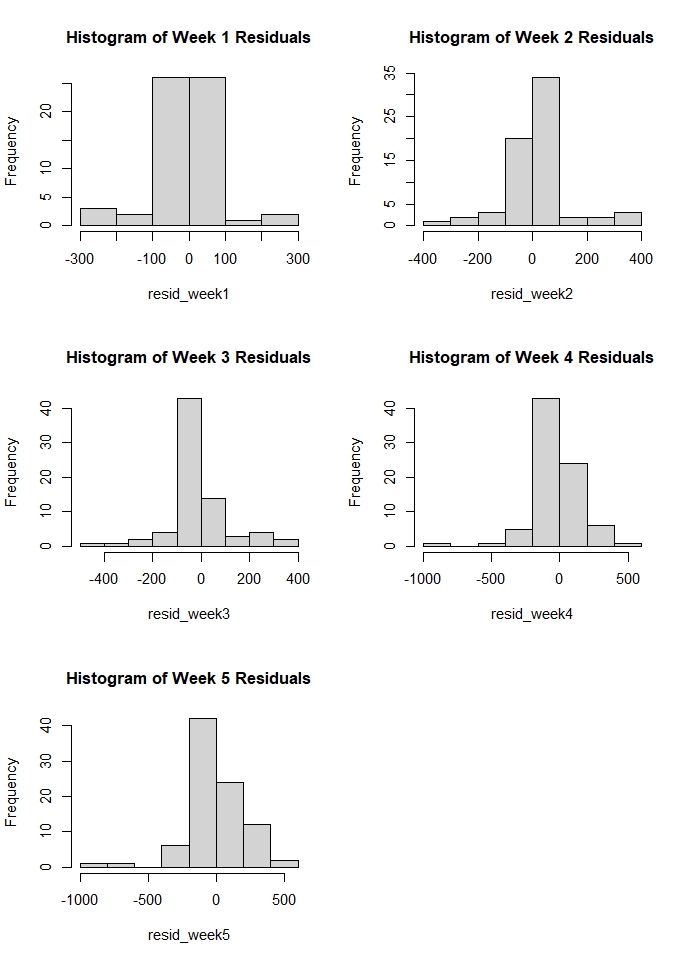


**Fig. S14.** Histogram of residual for autoregressive integrated moving average forecast model of Eid 2, 2023.


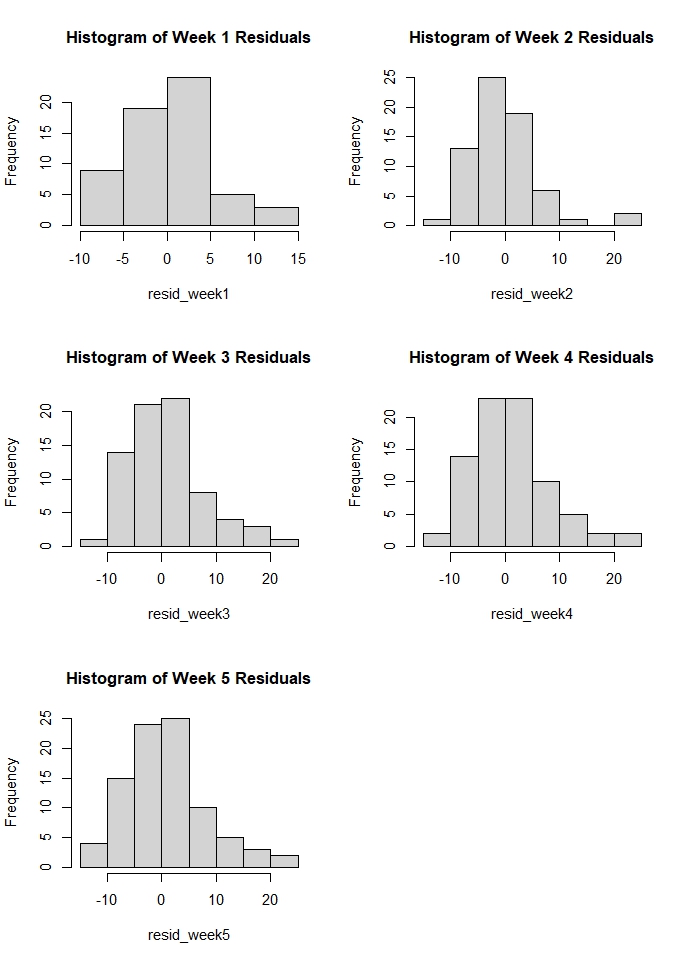


**Fig. S15.** Histogram of residual for autoregressive integrated moving average forecast model of Eid 1, 2024.

**Table S5**

Extended results of the autoregressive distributed lag models

| **Eid 1 (2023): ARDL (6,1,1,4)** | | | |
| --- | --- | --- | --- |
| Variables | Coefficient | t-statistic | Prob. |
| DC *_t-1_* | -0.57 | -4.73 | 0.00 |
| DC *_t-2_* | -0.33 | -2.43 | 0.02 |
| DC *_t-3_* | -0.38 | -2.76 | 0.01 |
| DC *_t-4_* | -0.31 | -2.33 | 0.02 |
| DC *_t-5_* | -0.19 | -1.41 | 0.16 |
| DC *_t-6_* | -0.30 | -2.56 | 0.01 |
| T | 0.08 | 0.91 | 0.36 |
| T *_t-1_* | 0.05 | 0.63 | 0.53 |
| H | -0.05 | -1.39 | 0.17 |
| H *_t-1_* | -0.06 | -1.67 | 0.10 |
| P | 0.08 | 1.03 | 0.31 |
| P *_t-1_* | 0.09 | 1.13 | 0.26 |
| P *_t-2_* | -0.11 | -1.32 | 0.19 |
| P *_t-3_* | -0.06 | -0.69 | 0.49 |
| P *_t-4_* | -0.03 | -0.33 | 0.74 |
| EI | 4.05 | 2.77 | 0.01 |
| **Eid 2 (2023): ARDL (10,2,1,8)** | | | |
| DC *_t-1_* | -0.48 | -3.85 | 0.00 |
| DC *_t-2_* | 0.11 | 0.86 | 0.39 |
| DC *_t-3_* | -0.20 | -1.62 | 0.11 |
| DC *_t-4_* | -0.10 | -0.90 | 0.37 |
| DC *_t-5_* | -0.35 | -3.00 | 0.00 |
| DC *_t-6_* | -0.46 | -3.58 | 0.00 |
| DC *_t-7_* | 0.38 | 2.76 | 0.01 |
| DC *_t-8_* | 0.21 | 1.46 | 0.15 |
| DC *_t-9_* | -0.13 | -0.95 | 0.35 |
| DC *_t-10_* | 0.13 | 0.97 | 0.34 |
| T | 0.02 | 0.11 | 0.92 |
| T *_t-1_* | 0.33 | 1.81 | 0.08 |
| T *_t-2_* | 0.08 | 0.48 | 0.63 |
| H | 0.11 | 1.15 | 0.26 |
| H *_t-1_* | 0.25 | 2.46 | 0.02 |
| P | 0.43 | 2.54 | 0.01 |
| P *_t-1_* | 0.29 | 1.50 | 0.14 |
| P *_t-2_* | -0.07 | -0.37 | 0.72 |
| P *_t-3_* | -0.03 | -0.17 | 0.87 |
| P *_t-4_* | -0.01 | -0.06 | 0.95 |
| P *_t-5_* | 0.22 | 1.12 | 0.27 |
| P *_t-6_* | 0.20 | 1.07 | 0.29 |
| P *_t-7_* | -0.42 | -2.36 | 0.02 |
| P *_t-8_* | -0.20 | -1.20 | 0.24 |
| EI | 20.23 | 4.05 | 0.00 |
| **Eid 1 (2024): ARDL (6,1,1,4)** | | | |
| DC *_t-1_* | -0.53 | -4.55 | 0.00 |
| DC *_t-2_* | -0.29 | -2.23 | 0.03 |
| DC *_t-3_* | -0.25 | -1.88 | 0.06 |
| DC *_t-4_* | -0.19 | -1.42 | 0.16 |
| DC *_t-5_* | -0.27 | -2.00 | 0.05 |
| DC *_t-6_* | -0.26 | -2.21 | 0.03 |
| T | 0.08 | 1.00 | 0.32 |
| T *_t-1_* | 0.04 | 0.47 | 0.64 |
| H | 0.05 | 1.04 | 0.30 |
| H *_t-1_* | 0.06 | 1.39 | 0.17 |
| P | 0.26 | 2.06 | 0.04 |
| P *_t-1_* | -0.17 | -1.28 | 0.21 |
| P *_t-2_* | 0.07 | 0.55 | 0.58 |
| P *_t-3_* | 0.07 | 0.55 | 0.59 |
| EI | 2.71 | 1.27 | 0.21 |
| *ARDL* autoregressive distributed lag; *DC* Dengue case; *T* temperature; *H* Humidity; *EI* Eid intervention. | | | |


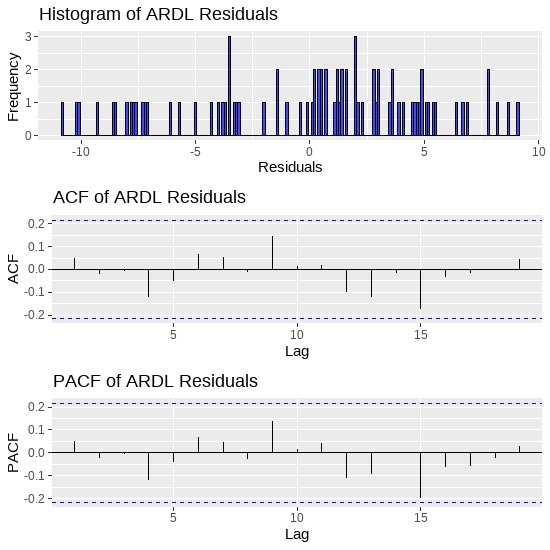


**Fig. S16.** Residuals, autocorrelation function, and partial autocorrelation function plots for the autoregressive distributed lag model of Eid 1, 2023.


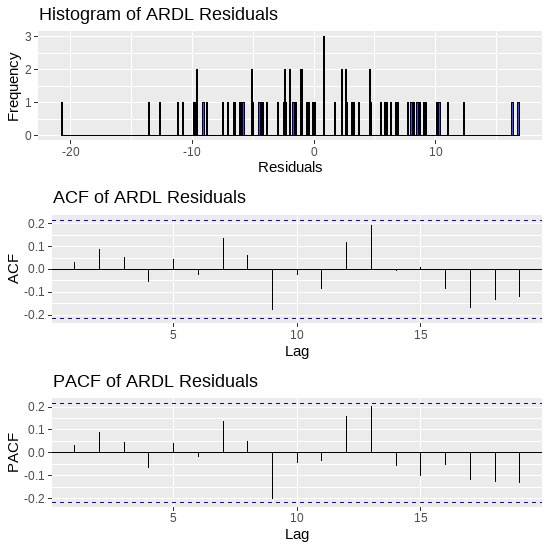


**Fig. S17.** Residuals, autocorrelation function, and partial autocorrelation function plots for the autoregressive distributed lag model of Eid 2, 2023.


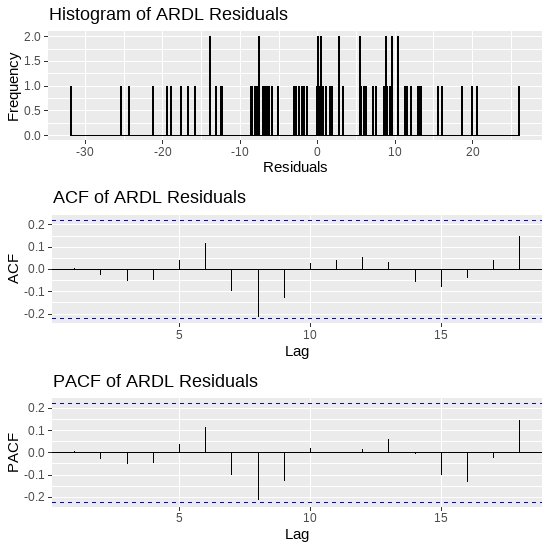


**Fig. S18.** Residuals, autocorrelation function, and partial autocorrelation function plots for the autoregressive distributed lag model of Eid 1, 2024.
